# Bidirectional epigenetic editing reveals hierarchies in gene regulation

**DOI:** 10.1038/s41587-024-02213-3

**Published:** 2024-05-17

**Authors:** Naomi M. Pacalin, Zachary Steinhart, Quanming Shi, Julia A. Belk, Dmytro Dorovskyi, Katerina Kraft, Kevin R. Parker, Brian R. Shy, Alexander Marson, Howard Y. Chang

**Affiliations:** 1 Center for Personal Dynamic Regulomes, Stanford University, Stanford, CA USA; 2 Department of Bioengineering, Stanford University, Stanford, CA USA; 3 Gladstone-UCSF Institute of Genomic Immunology, San Francisco, CA USA; 4 Department of Medicine, University of California, San Francisco, San Francisco, CA USA; 5 Department of Laboratory Medicine, University of California, San Francisco, San Francisco, CA USA; 6 Program in Epithelial Biology, Stanford University School of Medicine, Stanford, CA USA; 7 Cartography Biosciences, Inc., South San Francisco, CA USA; 8 UCSF Helen Diller Family Comprehensive Cancer Center, University of California, San Francisco, San Francisco, CA USA; 9 Parker Institute for Cancer Immunotherapy, San Francisco, CA USA; 10 Institute for Human Genetics, University of California, San Francisco, San Francisco, CA USA; 11 Department of Microbiology and Immunology, University of California, San Francisco, San Francisco, CA USA; 12 Diabetes Center, University of California, San Francisco, San Francisco, CA USA; 13 Innovative Genomics Institute, University of California, Berkeley, Berkeley, CA USA; 14 Howard Hughes Medical Institute, Stanford University, Stanford, CA USA

**Keywords:** Gene expression profiling, High-throughput screening, Epigenetics, Gene expression profiling, Gene regulation

## Abstract

CRISPR perturbation methods are limited in their ability to study non-coding elements and genetic interactions. In this study, we developed a system for bidirectional epigenetic editing, called CRISPRai, in which we apply activating (CRISPRa) and repressive (CRISPRi) perturbations to two loci simultaneously in the same cell. We developed CRISPRai Perturb-seq by coupling dual perturbation gRNA detection with single-cell RNA sequencing, enabling study of pooled perturbations in a mixed single-cell population. We applied this platform to study the genetic interaction between two hematopoietic lineage transcription factors, SPI1 and GATA1, and discovered novel characteristics of their co-regulation on downstream target genes, including differences in SPI1 and GATA1 occupancy at genes that are regulated through different modes. We also studied the regulatory landscape of *IL2* (interleukin-2) in Jurkat T cells, primary T cells and chimeric antigen receptor (CAR) T cells and elucidated mechanisms of enhancer-mediated *IL2* gene regulation. CRISPRai facilitates investigation of context-specific genetic interactions, provides new insights into gene regulation and will enable exploration of non-coding disease-associated variants.

## Abstract (web summary)

CRISPRai simultaneously activates and represses two genes in single cells.

## Main

Programmable epigenetic editing tools, specifically CRISPR activation (CRISPRa)^1–5^ and CRISPR interference (CRISPRi)^6,7^, are valuable for uncovering functional effects of genes and non-coding genetic elements, such as enhancers^8–15^. Dual CRISPR perturbations, in which two genes are perturbed simultaneously, are uniquely able to identify genetic interactions and epistasis, which, in turn, enables the rapid mapping of genetic pathways^16–20^. Previously, most large-scale dual gain-of-function and loss-of-function CRISPR perturbation screens employed CRISPR knockout (CRISPRko)^18,21–23^, but these approaches are limited in their ability to study multiplex perturbations and non-coding elements. CRISPRko introduces double-stranded DNA (dsDNA) breaks via Cas9 nuclease cutting, which triggers DNA damage pathways^24,25^ and can result in indels^26,27^ and structural rearrangements^28,29^. Furthermore, CRISPRko has the potential for forming regulatory landscapes via introduction of transcription factor (TF) binding sites or reduction in distance between existing regulatory elements (REs), as well as the potential for inadequately perturbing REs, such as enhancers, for which small indels may not alter function. Multiplexed CRISPRi can address non-coding element epistasis^30^ but may be limited to elements that are contemporaneously active in the cell type being studied. More recently, methods for bidirectional perturbations of two loci simultaneously, including paired CRISPRa and CRISPRi, have been developed but have been applied only to non-mammalian cells, are transient or are targeted to only a few genes^31–39^. New tools are needed that are compatible with studying genetic interactions in human cells, pooled high-throughput single-cell readouts and multiplexed bidirectional control of non-coding elements and are highly scalable to hundreds or thousands of perturbations. Epigenetic perturbations are key for studying functional effects of non-coding elements such as enhancers in their endogenous locus because enhancer functionality is likely mediated through structural chromatin contacts, histone modifications, TF requirement and other effects^40–47^. Furthermore, comprehensive investigation of genetic interactions requires versatile bidirectional perturbation tools in addition to existing unidirectional tools to study the complete range of context-specific genetic interactions^8,48–50^.

Additionally, the power of high-throughput and high-content readouts has been well demonstrated. Perturb-seq, a method for single-cell transcriptome profiling coupled with CRISPR guide RNA (gRNA) readout^51–55^, enables investigation of gene networks^51–53^ and disease risk genes^56^. Previous Perturb-seq methods have been limited to a single perturbation type (that is, CRISPRa, CRISPRi or CRISPRko), and current methods cannot perform combinatorial bidirectional perturbations.

To broaden the toolkit for studying genes and non-coding elements and to enable investigation of context-specific genetic interactions, we developed CRISPRai, a system for bidirectional epigenetic editing of two loci simultaneously in a single cell. We use orthogonal activating (CRISPRa) and repressive (CRISPRi) perturbations to perturb two distinct genomic loci simultaneously. We activate one element and repress another to study how pairs of genetic elements functionally interact, and we apply this tool to study genes and enhancers. First, we developed dual-gRNA-capture CRISPRai Perturb-seq and applied it to study interactions between genes. We investigated the genetic interaction between *SPI1* (Spi-1 proto-oncogene) and *GATA1* (GATA1 binding protein 1)^57–60^, two well-characterized lineage-directing TFs for the myeloid (SPI1) and erythroid (GATA1) lineages. We found that bidirectional perturbation enabled modulation of cell lineage signatures and enabled heightened perturbation phenotypes compared to single perturbations, and different TF occupancy relationships at downstream target genes resulted in different patterns of co-regulation. Second, we applied CRISPRai to investigate how multiple enhancers interact to regulate expression of a shared target gene, using the *IL2* (interleukin-2) gene in activated Jurkat T cells as a model system. We extended our findings from CRISPRai to primary human T cells using CRISPRi perturbations. We integrated our CRISPRai findings with epigenomic datasets to jointly assess function, chromatin accessibility, histone modifications, TF motif enrichment and chromatin looping. These integrated analyses revealed the existence of strong functional ‘gatekeeper’ enhancers that heavily compete with the promoter for transcriptional control and highlighted two main modes of regulation by gatekeeper enhancers: activity driven and contact driven. Overall, CRISPRai reveals insights into genetic interactions for both genes and non-coding elements and broadens the toolkit for investigating the functional effects of the genome.

## Results

### CRISPRai system for bidirectional epigenetic editing

**Fig. 1:**
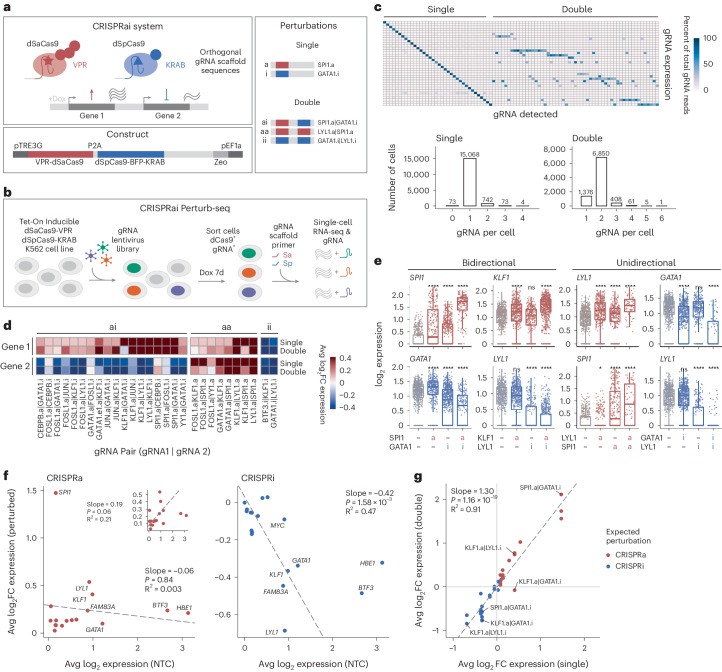
CRISPRai system for bidirectional epigenetic editing in individual cells. **a**, Schematic of CRISPRai system (top), CRISPRai construct (bottom) and CRISPRai perturbations (right). **b**, Schematic of dual-gRNA CRISPRai Perturb-seq screen in K562 cell line. **c**, gRNA expression (rows) by gRNA detected (columns). Bar plot shows the number of gRNA per cell detected in all cell–gRNA expression pairs passing a threshold. **d**, Average log_2_FC gene expression for each pair of CRISPRai target genes (columns) in cells receiving either a single or double perturbation (rows). Gene expression for gene 1 (top) and gene 2 (bottom) from the pair is shown. **e**, Examples of average log_2_FC gene expression in single and double perturbations for indicated gene pairs with ai, aa or ii perturbations. **f**, Correlation between perturbation strength and baseline target gene expression level for CRISPRa (left) and CRISPRi (right). **g**, Correlation between perturbation strength in single versus double perturbations for a given gene, labeled with double perturbation received. **d**–**g**, DE tests performed relative to cells with NTC gRNAs. All gRNA groups included have *n* > 40 (**d**–**f**,**h**) and *n* > 20 (**g**) cells. **e**, *n* = 73–600. Box plot, median and interquartile range (IQR). Box whiskers, 1.5× IQR. Two-sided Wilcoxon test. **f**,**g**, Linear regression. Significance cutoffs: NS *P* > 0.05, *P ≤ 0.05, **P ≤ 0.01, ***P ≤ 0.001, ****P ≤ 0.0001. NS, not significant.

**Extended Data Fig. 1:**
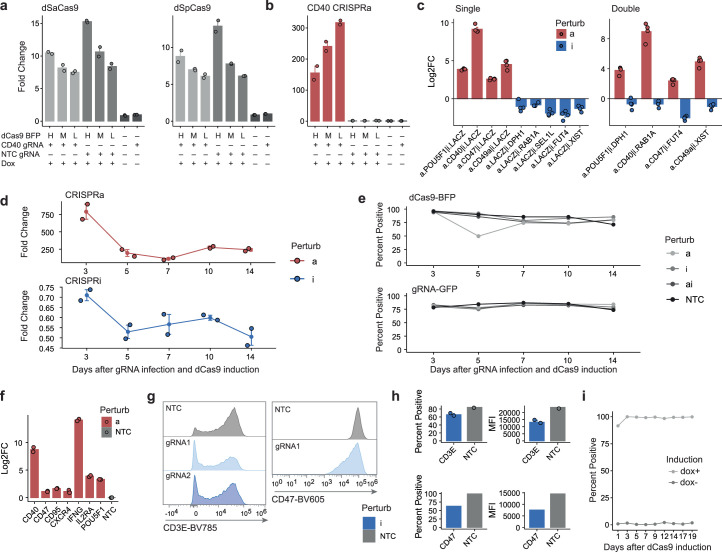
(related to Figs. 1, 3). Development of CRISPRai system. **(a-e)** Development of K562 CRISPRai cell line in bulk. **(a,b)** Construct expression, inducibility of CRISPRai system, and dependence on dCas9 cassette expression level. Expression level by qPCR of (a) dSaCas9 and dSpCas9, and (b) CD40 gene after sorting high, mid, low (H,M,L) dCas9 expression levels by FACS. Cells express gRNA for CD40 CRISPRa or NTC. **(c)** Log2FC gene expression by qPCR for several genes in single and bidirectional perturbations. **(d)** Time course of gene expression by qPCR during CRISPRai single perturbations of 2 genes for CRISPRa (top) and CRISPRi (bottom). **(e)** Time course of construct expression by flow cytometry of dCas9 cassette (top) and gRNA (bottom). **(d,e)** Perturbation effect and dCas9 expression remains high for >14 days, dCas9 is not silenced. **(f-i)** Development of Jurkat CRISPRai cell line in bulk. **(f)** Log2FC gene expression by qPCR of several genes in CRISPRa single perturbations. **(g)** Protein levels by flow cytometry during CRISPRi single perturbations. **(h)** Same data as (g). **(i)** Time course of dox induction by flow cytometry showing dCas9 cassette does not silence for >20 days. **(a-d,f)** All qPCR analysis was done using the double delta Ct method relative to housekeeping gene *ACTB* and NTC gRNAs, n = 4 (2 biological replicates, 2 technical replicates). **(g-h)** n = 1 or 2 (1 or 2 gRNAs). NTC gRNA is against *lacZ* gene. **(a-d,f,h)** Data are mean ± SEM.

We developed a system for bidirectional epigenetic editing (CRISPRai) that enables activation and repression of two distinct loci simultaneously in a single cell and can be applied to both genes and enhancers (Fig. 1a and Extended Data Fig. 1a–i). Our system comprises Tet-On doxycycline (dox)-inducible CRISPRa and CRISPRi machinery and leverages two orthogonal species of catalytically dead Cas9 (dCas9). We express activator-fused dCas9 from *Staphylococcus aureus* (VPR-dSaCas9) and repressor-fused dCas9 from *Streptococcus pyogenes* (dSpCas9-KRAB, *ZNF10* or *KOX1* domain) simultaneously to achieve species-specific recognition where two distinct gRNA scaffold sequences pair with their cognate dCas9 (refs. ^61,62^). This enables two distinct perturbations at two different loci in the same cell at the same time (Fig. 1a and Extended Data Fig. 1a–i). After generating stable K562 (Extended Data Fig. 1a–e) and Jurkat (Extended Data Fig. 1f–i) CRISPRai cell lines, we validated the system using bulk assays. We confirmed construct expression, robust induction by dox and tunable control of CRISPR perturbation strength based on dCas9 expression level (Extended Data Fig. 1a,b). Bidirectional double perturbations were similar in strength to the respective single perturbations (ranging from −3 to +13 log_2_ fold change (FC) in gene expression; Extended Data Fig. 1c,f–h). Finally, we confirmed stable expression of both dCas9 and the gRNA over 14–20 d (Extended Data Fig. 1d,e,i).

**Extended Data Fig. 2:**
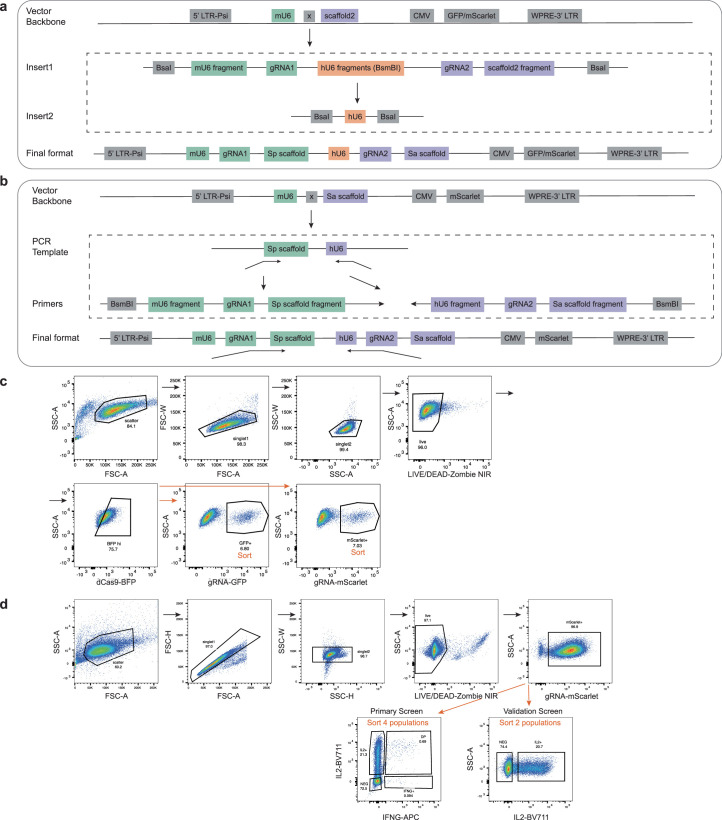
(related to Figs. 1–6). Dual CRISPR gRNA cloning and gating strategy for screens. **(a,b)** Cloning strategy for: (a) dual gRNAs for K562 Perturb-seq screen, and (b) dual gRNAs for Jurkat gRNA enrichment screen. **(c,d)** Gating strategy for: (c) K562 Perturb-seq screen and (d) Jurkat gRNA enrichment screen, including dCas9 cassette (BFP) and gRNA constructs (GFP or mScarlet).

**Extended Data Fig. 3:**
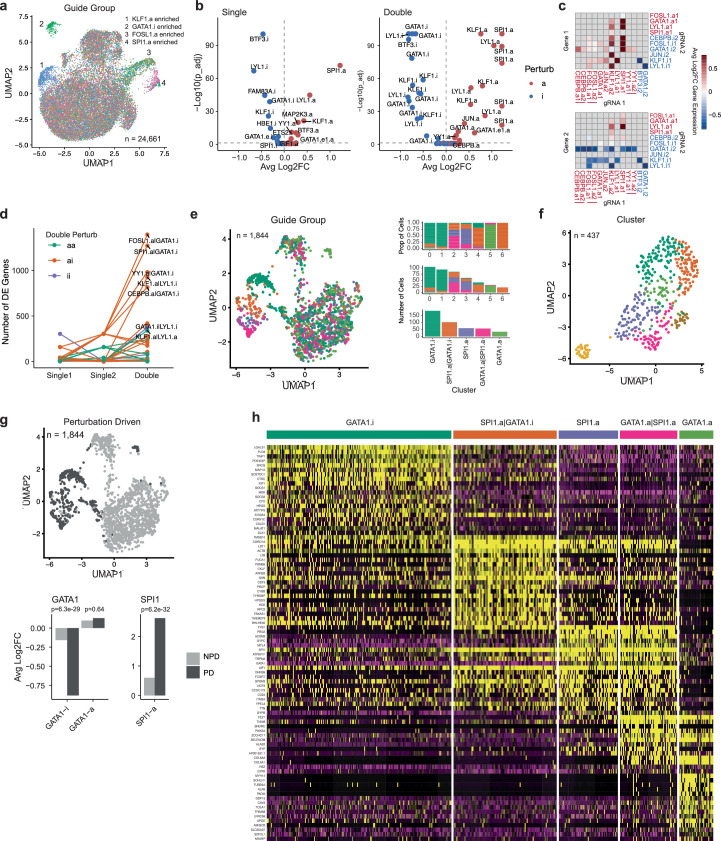
(related to Fig. 1). Single-cell K562 CRISPRai screen and SPI1 and GATA1 quality control. **(a)** Visualization of all single-cell transcriptomes from Perturb-seq. Each dot represents one cell which, colored by detected gRNA or gRNA pair. Labeled clusters highlight cells with strong perturbation-driven phenotypes. **(b)** Magnitude and significance of CRISPRai target gene DE in single (left) and double (right) perturbations. Horizontal dotted line at p_adj ≤ 0.05. **(c)** Average log2FC expression for all double perturbations included in the screen. Axes show gRNA 1 and gRNA 2 in the double perturbation pair. All pairs with n > 30 cells shown. **(d)** Number of DE genes in single and double perturbations for all perturbation sets. All cells, including non-perturbation driven cells, are included for DE testing. **(e)** Visualization of cells with gRNA calls in the SPI1-GATA1 perturbation set. Proportion or number of cells per gRNA group is shown. **(f)** Visualization of cells from (e) in the perturbation-driven cell subset. **(g)** Same data as (f), colored by perturbation-driven (PD) or non-perturbation driven (NPD) clustering (top), and average log2FC gene expression of *GATA1* and *SPI1* in PD and NPD cells. Perturbation-driven cells were defined as cells in all clusters that do not comprise a mixture of all gRNA groups. **(g,h)** Logistic regression. **(h)** Top DE genes. Log2FC and DE gene testing is relative to NTC, logistic regression was used for DE. Data from gRNA groups with (a,b) n > 20, (d) n > 50 cells per group. Significance cutoffs: ns p > 0.05, * p ≤ 0.05, ** p ≤ 0.01, *** p ≤ 0.001, **** p ≤ 0.0001.

We next developed dual perturbation direct gRNA capture Perturb-seq, or CRISPRai Perturb-seq, to study gene–gene interactions with a single-cell transcriptome readout in K562 cells. We designed 82 single (42 CRISPRa and 40 CRISPRi), 22 double (18 bidirectional pairs and four unidirectional pairs as controls) and 12 non-targeting control (NTC) gRNAs containing selected combinations of single and double perturbations against a panel of 19 lineage-relevant TFs, chromatin remodelers and proto-oncogenes, with two gRNAs per gene (Fig. 1b, Extended Data Fig. 2a and Supplementary Tables 1 and 2). We used the single perturbations to evaluate gRNA efficacy for CRISPRa versus CRISPRi. To detect gRNAs in single-cell sequencing data, we extended recently developed methods of droplet-based direct gRNA sequence detection for CRISPRai^63,64^. We spiked in two oligos complementary to each gRNA scaffold region into the reverse transcription (RT) reaction. We captured a total of 24,661 cells (14,086 cells with single perturbations, 6,631 cells with double perturbations and 3,944 cells with NTCs). Single and double perturbations were performed using separate gRNA pools in separate single-cell captures, and sequencing data from all captures were combined for analysis (Extended Data Figs. 2c and 3a–d). To determine gRNA detection efficacy, we assessed the number of gRNA counts per cell. We found that 94.4% of cells expected to have single perturbations had one gRNA assigned and 78.7% of cells expected to have double perturbations had two gRNAs assigned (Fig. 1c and Methods). Twenty-one of 22 designed double perturbations (95.5%) were detected.

We investigated the CRISPRai perturbation strength and directionality across the target genes present in our pool. The system enabled consistent bidirectional expression changes for both target genes in all double perturbations, with the log_2_FC gene expression increasing or decreasing as expected in each condition (range from −1.08 to +2.11 gene expression log_2_FC; Fig. 1d,e and Extended Data Fig. 3b,c). In addition to bidirectional perturbations, the CRISPRai system also allows for unidirectional dual CRISPRaa and CRISPRii perturbations (Fig. 1a). We demonstrated the expected behavior for unidirectional CRISPRaa and CRISPRii combinations (Fig. 1d). The expression changes were statistically significant in both the single and double perturbations and spanned a range of log_2_FC (Fig. 1d and Extended Data Fig. 3b). We found that different genes had variable susceptibility to perturbation. For example, *SPI1* was highly responsive to activation but not repression, whereas the opposite was true for *GATA1* (Fig. 1e and Extended Data Fig. 3b). Finally, multiple independent gRNAs targeting the same gene had concordant impacts on target gene expression (Extended Data Fig. 3c).

We next investigated the aggregate characteristics of bidirectional epigenetic editing across all of the genes in the pool. Baseline gene expression was inversely correlated with perturbation strength for CRISPRi (R^2^ = 0.47, *P* = 1.58 × 10^−3^, slope = −0.42; Fig. 1f, right). In contrast, baseline gene expression and strength of CRISPRa did not have a clear relationship (R^2^ = 0.003, *P* = 0.84, slope = −0.06; Fig. 1f, left). Furthermore, perturbation strength was highly correlated between single and double perturbations (log_2_FC target gene expression: R^2^ = 0.91, *P* ≤ 1.16 × 10^−19^, slope = 1.30; Fig. 1g). This confirms the orthogonality of the two dCas9 species and indicates that CRISPRai dual perturbations do not dilute the perturbation strength of the individual perturbations in the pair^65–67^. Overall, CRISPRai enables robust, scalable and bidirectional interrogation of diverse target genes.

### CRISPRai reveals context-specific genetic interactions

**Fig. 2:**
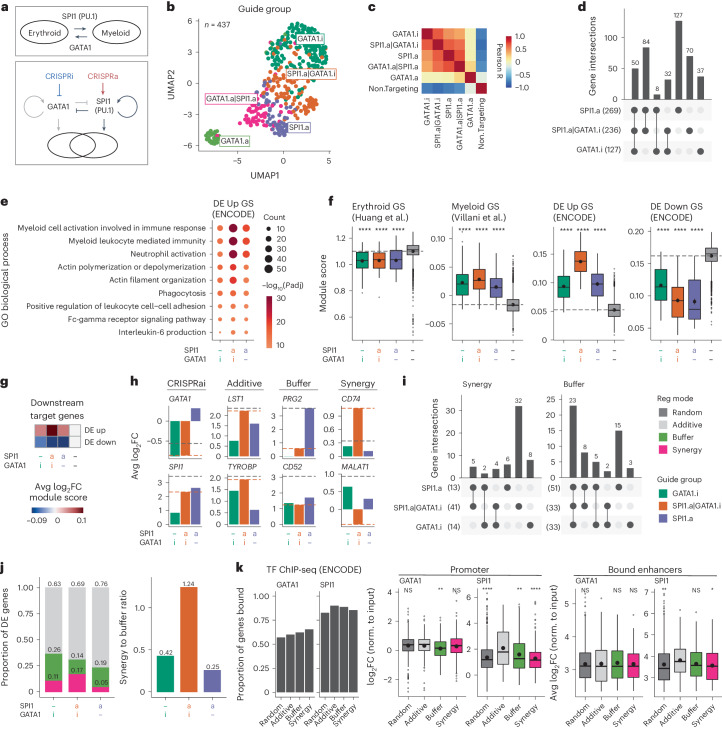
CRISPRai reveals context-specific genetic interaction for SPI1 and GATA1. **a**, Schematic of SPI1−GATA1 genetic interaction. **b**, Visualization of perturbed K562 cells. Each dot represents one cell, colored by detected gRNA or gRNA pair. **c**, Pearson correlation of normalized and centered single-cell transcriptomes over all genes. **d**, Overlap of DE genes. **e**, Biological process GO term enrichment for DE genes upregulated in perturbed cells relative to NTC, selected terms. **f**, Module scores for indicated gene sets. Gene set sizes from left to right: *n* = 419, 394, 5,190 and 6,003. **g**, Same data as **f**, showing module scores in log_2_FC. **h**, Average log_2_FC gene expression of *SPI1* and *GATA1* and selected ENCODE annotated downstream target genes. Dashed line: expected additive model (gray) and observed bidirectional perturbation (orange). Additive (observed = expected), synergy (observed > expected or opposite sign) and buffer (observed < expected). **i**, Overlap of synergy and buffer gene sets with DE gene sets; number indicates gene set size. **j**, Proportion of DE genes under each regulatory mode (left) and ratio of number of genes under synergistic and buffering regulation (right). **k**, TF occupancy at synergy and buffer gene sets, showing proportion of genes with one or more RE bound by GATA1 or SPI1 including promoter (within 1 kb) or any ABC model^45^ predicted enhancer (left) and log_2_FC of promoter or average log_2_FC within enhancers (right). Includes all annotated genes with non-zero ChIP-seq reads. Each dot is the average signal across all bound REs for one gene. Additive set: subset of 50 genes with most additive phenotype. Gene set sizes from left to right: *n* = 300, 50, 53 and 55. *n* = 141–900 (two biological replicates, one additional technical replicate). **d**–**g**,**i**–**k**, Significance cutoffs for DE genes are abs(log_2_FC) > 0.5, *P*_adj < 0.05; DE gene testing for each gRNA group is against NTC. All gRNA groups have *n* > 34 (**b**,**c**) and *n* > 59 (**d**–**k**) cells. Logistic regression was used for DE gene testing. **e**, One-sided Fisher’s exact test. **f**,**k**, Box plot, median and interquartile range (IQR). Box whiskers, 1.5× IQR. Two-sided Wilcoxon test. Significance cutoffs: NS *P* > 0.05, *P ≤ 0.05, **P ≤ 0.01, ***P ≤ 0.001, ****P ≤ 0.0001. GS, gene set; norm., normalized; NS, not significant.

Pairwise CRISPR perturbations can identify genetic interactions between genes^16–20,23^, and CRISPR screens with single-cell readouts enable investigation of the global regulatory effects of a given gene, including identification of downstream target genes and regulatory gene modules controlled by the perturbed gene^51–55,64^. Thus, we next applied CRISPRai to investigate genetic interactions. By analyzing our K562 CRISPRai Perturb-seq data, we identified the SPI1−GATA1 genetic interaction as an excellent example of the ability of CRISPRai to reveal new insights into TF biology (Fig. 2a). Thus, the design of the initial CRISPRai screen allowed us to rigorously benchmark our double perturbations as well as to investigate the SPI1−GATA1 genetic interaction in more detail.

SPI1 and GATA1 are pivotal hematopoietic TFs that are essential for myeloid and erythroid lineage development, and they are known to interact and inhibit each other’s function^57–60^ (Fig. 2a). We first investigated the global transcriptome-wide effects of all combinations of SPI1 and GATA1 perturbations included in the screen in 437 cells. After clustering and dimensionality reduction of the single-cell RNA sequencing (scRNA-seq) data, we found that the perturbed cells clustered according to the detected gRNAs (Fig. 2b). Furthermore, double perturbations were between the corresponding single perturbations in the low-dimensional uniform manifold approximation and projection (UMAP) visualization, demonstrating a gradient in transcriptomic signature resulting from the perturbations, which was also apparent via correlation analysis (Fig. 2b,c and Extended Data Fig. 3e–h).

**Extended Data Fig. 4:**
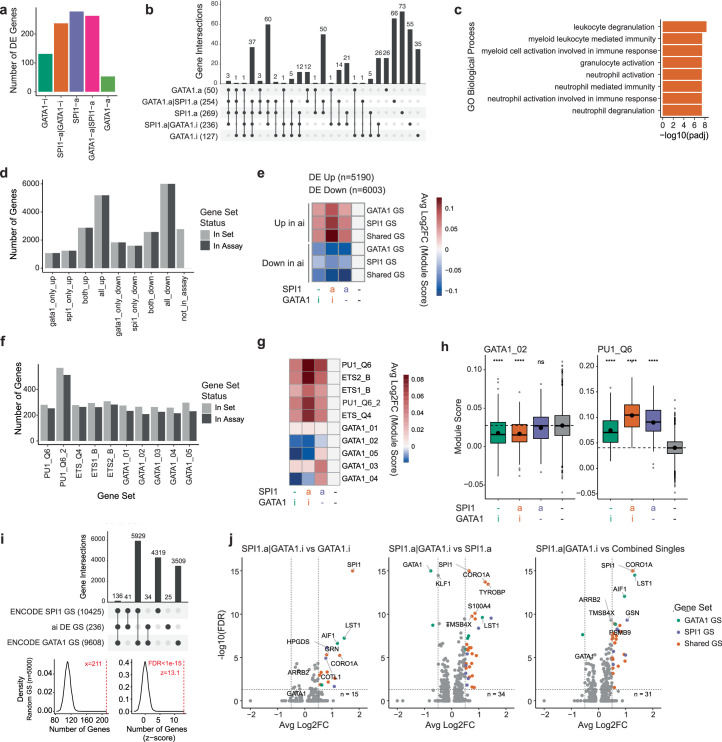
(related to Fig. 2). Additional analysis for SPI1 and GATA1 genetic interaction. **(a)** DE genes by gRNA group. **(b)** Overlap of DE genes across gRNA groups. **(c)** Biological process GO term enrichment for upregulated genes uniquely DE in SPI1.a|GATA1.i bidirectional perturbation. **(d)** Gene set size for ENCODE TF target gene sets^70–72^ and the subset that is present in the dataset for GATA1 only, SPI1 only, or shared TF target genes grouped by up or down regulation in the SPI1.a|GATA1.i bidirectional perturbation. **(e)** Average log2FC gene expression module score for gene sets in (d). **(f)** Gene set sizes for MSigDb TF target gene sets^73,74^ and the subset that is present in the dataset for SPI1 (PU.1), GATA1, or ETS family gene sets. **(g)** Average log2FC gene expression module score for gene sets in (f). **(h)** Examples from (g). Gene set sizes from left to right: n = 265, 280. **(i)** Overlap between statistically significant DE genes in the bidirectional perturbation and SPI1 and GATA1 ENCODE TF target gene sets (top), and significance of overlap between CRISPRai DE genes and ENCODE gene sets relative to null distribution (bottom). **(j)** DE genes in bidirectional perturbation relative to individual or combined single perturbations, colored by ENCODE TF target gene set. Only genes in ENCODE TF target genes sets were tested for DE. **(a-c,i-j)** Significance cutoffs for DE genes are: abs(log2FC) > 0.5, p_adj < 0.05. Log2FC and DE gene is relative to NTC, logistic regression was used for DE gene testing. All gRNA groups have (a,b) n > 34 and (c-j) n > 59 cells. **(c)** One-sided Fisher’s exact test, BH correction. **(h)** Boxplot, median and IQR. Box whiskers, 1.5x IQR. Two-sided Wilcoxon test. Significance cutoffs: ns p > 0.05, * p ≤ 0.05, ** p ≤ 0.01, *** p ≤ 0.001, **** p ≤ 0.0001.

Next, we prioritized the SPI1.a|GATA1.i bidirectional double perturbation and its corresponding single perturbations for further analysis, due to the responsiveness of each gene to CRISPRa and CRISPRi (the SPI1.a|GATA1.i perturbation is referred to below as ‘bidirectional perturbation’). The set of differentially expressed (DE) genes (relative to NTC gRNAs) in the SPI1.a|GATA1.i bidirectional perturbation was composed of two groups of genes that were shared by the corresponding single perturbations (SPI1.a or GATA1.i) and a third bidirectional perturbation-specific group of 70 genes (Fig. 2d and Extended Data Fig. 4a,b). The upregulated DE genes for each perturbation condition were enriched for relevant biological process Gene Ontology (GO) terms, including myeloid cell activation, actin polymerization, cell adhesion, phagocytosis and other immune signaling pathways, with the bidirectional perturbation being most significantly enriched (Fig. 2e). DE genes specific to the bidirectional perturbation were similarly enriched for relevant processes (Extended Data Fig. 4c).

We next asked if the CRISPRai perturbation modulated expression of known downstream target genes of SPI1 and GATA1 (Fig. 2a). Because SPI1 and GATA1 exhibit opposing and antagonistic effects on the myeloid and erythroid lineages, we hypothesized that known downstream target gene sets would have heightened gene expression changes in the bidirectional perturbation relative to the single perturbations. We investigated two gene sets from the literature: erythroid marker genes (*n* = 419)^68^ and myeloid marker genes (*n* = 394)^69^. As expected, the erythroid gene signature decreased and the myeloid gene signature increased in both the single and bidirectional perturbations, with the myeloid signature being most extreme in the bidirectional perturbation (Fig. 2f). Additionally, we used the set of annotated target genes for these two TFs from ENCODE^70–72^ and grouped the gene sets based on upregulation or downregulation in the bidirectional perturbation. As expected, the average expression of known target genes was more extreme in the bidirectional perturbation than the single perturbations (Fig. 2f,g). This pattern persisted after grouping the gene sets based on identity of TF regulator: GATA1 only, SPI1 only or shared (Extended Data Fig. 4d,e). We validated this regulatory pattern on gene sets from a different database (Molecular Signatures Database)^73,74^ and saw similar results (Extended Data Fig. 4f–h). Additionally, we confirmed that the set of statistically significant DE genes in the bidirectional perturbation was highly overlapping with annotated SPI1 and GATA1 target gene sets (Extended Data Fig. 4i,j).

We then used the bidirectional perturbation data to identify downstream target genes that were nonlinearly regulated by SPI1 and GATA1. We used an additive model of gene regulation that has previously been used for pairwise CRISPR perturbations^52,53,75^. First, we classified DE genes as belonging to synergistic, buffering or additive modes of regulation (Fig. 2h and Supplementary Table 3). The largest group of genes classified as being under synergistic regulation was unique to the bidirectional perturbation DE gene set (56.1%), highlighting the ability of CRISPRai to provide new insights into cooperation between TFs. As expected, the largest group of genes classified as being under buffering regulation was shared across the DE gene sets of the three perturbation groups (41.1%) (Fig. 2i). We then compared the proportions of each regulatory mode for DE genes across perturbations. For each perturbation, most genes were under additive regulation (63–76%) (Fig. 2j, left). Synergistic regulation (5–17%) was less common than buffering regulation (14–26%). To compare across the three perturbation groups, we accounted for differences in DE gene set sizes by calculating the ratio between the numbers of synergistic and buffering genes. This ratio was greatest for the bidirectional perturbation (bidirectional perturbation 1.24 versus single perturbations 0.42 and 0.25), which demonstrates that CRISPRai enables identification of genes under synergistic regulation that would be missed by studying only single perturbations (Fig. 2j, right).

We then sought to further investigate the synergistic and buffering genes and provide insight into the mechanism underlying the different modes of gene regulation observed. We compared the SPI1 and GATA1 occupancy profiles for the buffering, additive and synergistic gene sets. We calculated the log_2_FC chromatin immunoprecipitation followed by sequencing (ChIP-seq) signal of SPI1 and GATA1 (ENCODE data^70,71^) within 1 kb of the promoter or within predicted enhancers for a given gene. The set of predicted enhancers was generated from the activity-by-contact (ABC) model^44,45^. We found that additive genes were enriched for genes occupied by both SPI1 and GATA1 (Fig. 2k, left). Synergistic genes had decreased SPI1 occupancy at the promoter and enhancers relative to additive and buffering genes but had similar GATA1 occupancy as additive genes (Fig. 2k, right). This suggests that synergistic genes may have higher dose sensitivity due to an imbalance in binding of these two TFs. Conversely, buffering genes had decreased occupancy of both SPI1 and GATA1 at the promoter (Fig. 2k, right). The correlated occupancy of these two TFs at buffering genes suggests that binding of one TF may influence the other. In summary, CRISPRai enables the investigation of important TFs and provides insight into how these TFs interact to regulate overlapping downstream gene modules.

### CRISPRai defines enhancer–promoter regulatory hierarchies

After demonstrating the utility of the CRISPRai system for investigating *trans*-regulatory effects and gene–gene interactions, we extended our method to investigate *cis*-regulatory effects by studying enhancer–promoter and enhancer–enhancer interactions (denoted enhancer–transcription start site (E–TSS) and E–E, respectively). Previous studies showed that enhancer impact on target gene expression is governed by several factors, including distance to TSS and enhancer strength, and that some enhancers may have redundant function^40,47,76,77^. However, it is unknown how multiple enhancers may interact to control target gene expression or how enhancers interact differentially with the TSS. We applied CRISPRai to study the regulatory landscape of the *IL2* and *IFNG* (interferon-gamma) genes to investigate these questions. We focused on the *IL2* regulatory landscape due to its more interesting regulatory landscape.

**Fig. 3:**
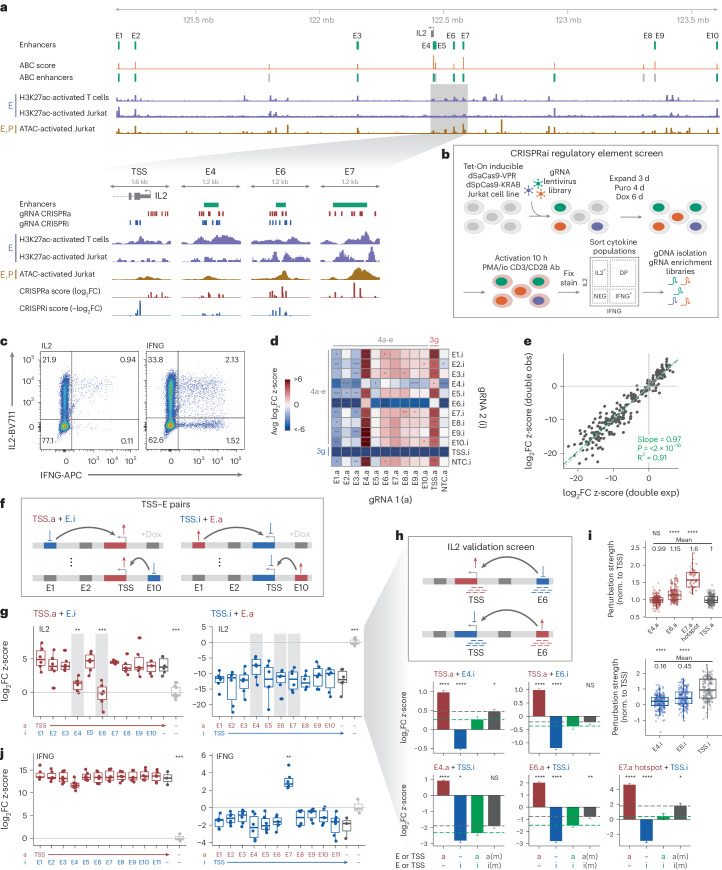
CRISPRai defines hierarchies in transcriptional regulation between promoter and enhancers. **a**, Genome tracks showing regulatory landscape of *IL2* gene locus for primary T cells and Jurkat T cells. Insets show data for selected enhancers, including gRNA CRISPRa score (log_2_FC) and CRISPRi score (−log_2_FC). **b**, Schematic of CRISPRai RE screen in Jurkat T cells. **c**, Intracellular cytokine staining in activated Jurkat. **d**, Average log_2_FC z-score (IL2^+^/NEG of all single, bidirectional and NTC gRNA pairs. Two gRNAs per enhancer (2 a, 2 i). RE hierarchy demonstrated when one perturbation overrides the expected effect of a second perturbation (for example, TSS.i bidirectional perturbations result in similar effect as TSS.i single perturbations; note that E6.i overrides other E.a). Results of specific columns and rows are expanded in subsequent figure panels. **e**, log_2_FC z-score (IL2^+^/NEG) for TSS–E bidirectional perturbations, showing expected and observed. **f**, Schematic of bidirectional TSS–E perturbation pairs. **g**, log_2_FC z-score (IL2+/NEG) for *IL2* gene for TSS–E gRNA pairs. **h**, Schematic of validation screen, eight gRNAs per enhancer (top) and examples of selected TSS–E pairs highlighted in **g** with gray bars, showing log_2_FC z-score (IL2^+^/NEG) (bottom). Bins represent single (a or i), bidirectional (ai) and expected bidirectional perturbation from additive model (ai model, gray); dashed lines show observed and expected bidirectional perturbations. Data are mean ± s.e.m. **i**, Perturbation strength, normalized to TSS perturbation, for selected enhancer single perturbations in the IL2 validation screen, mean annotated. **j**, log_2_FC z-score (IFNG^+^/NEG) for *IFNG* gene for TSS–E gRNA pairs. **d**,**e**,**g**, Data from *IL2* locus initial screen, *n* = 6 (three biological replicates, two gRNAs per enhancer). **j**, Data from *IFNG* locus screen, *n* = 6 (three biological replicates, two gRNAs per enhancer). **a**,**h**,**i**, Data from *IL2* locus validation screen, *n* = 147–168 for E4 and E6 pairs, *n* = 42 for E7 hotspot (three biological replicates, 7–8 gRNAs per enhancer, E7 hotspot derived from two gRNAs in E7). Significance was tested relative to TSS single perturbation (**g**,**i**,**j**) and observed bidirectional perturbation (**h**). **g**,**i**,**j**, Box plot, median and interquartile range (IQR). Box whiskers, 1.5× IQR. **d**,**h**,**i**, Two-sided Wilcoxon test. **d**, Benjamini–Hochberg correction. **e**, Linear regression. **g**,**j**, Two-sided *t*-test. Significance cutoffs: NS *P* > 0.05, *P ≤ 0.05, **P ≤ 0.01, ***P ≤ 0.001, ****P ≤ 0.0001. exp, expected; DP, double positive; NEG, negative; NS, not significant; obs, observed; Puro, puromycin.

**Extended Data Fig. 5:**
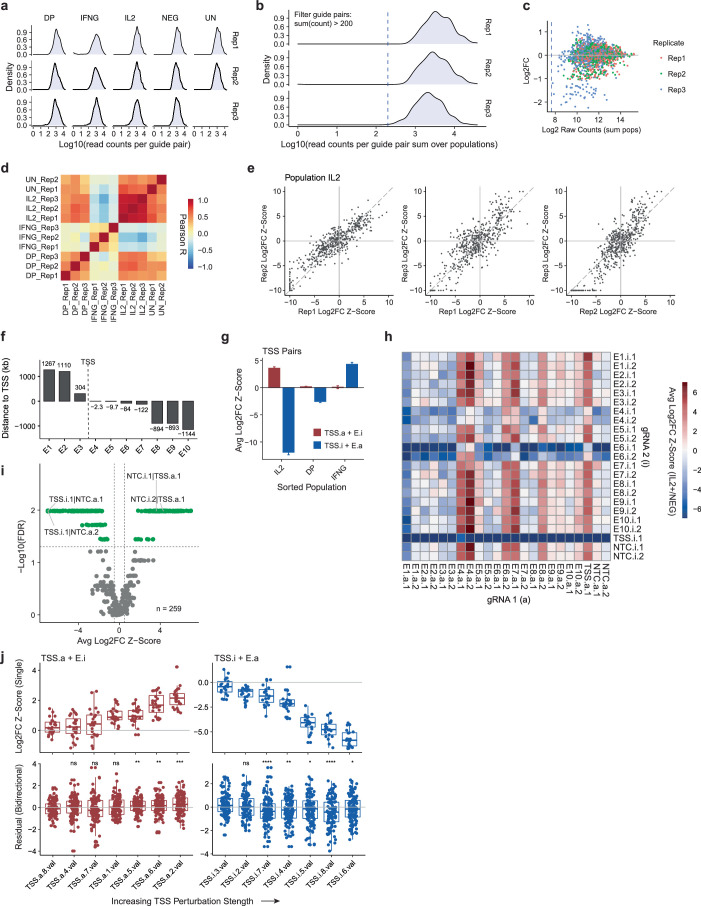
(related to Fig. 3). Additional data from Jurkat *IL2* locus initial screen and TSS-E perturbations. **(a)** Log10 raw read counts per gRNA pair by sorted population and biological replicate. DP = IL2 + IFNG+ (double positive), IFNG = IL2-IFNG + , IL2 = IL2 + IFNG-, NEG = IL2-IFNG- (double negative), UN=unsorted. **(b)** Log10 raw read counts per gRNA pair summed over all populations, with read count cutoff (blue dotted line) used for filtering reads. **(c)** Log2FC (IL2+/NEG) versus log2 raw read counts per gRNA, colored by biological replicate. **(d)** Pearson correlation of log2FC (IL2+/NEG) across all gRNAs for sorted populations and biological replicates. **(e)** Log2FC z-score (IL2+/NEG) correlation of biological replicates for IL2 sorted population. **(f)** Distance between enhancer midpoint and *IL2* TSS. **(g)** Average log2FC z-score (IL2+/NEG) for all single and double TSS-E pairs. Data are mean ± SEM. **(h)** Average log2FC z-score (IL2+/NEG) for all single, double, and NTC gRNA pairs. Columns and rows indicate gRNA1 and gRNA2 in the pair. **(i)** Magnitude and statistical significance for all gRNA pairs, including single, bidirectional, and NTC gRNAs. TSS single perturbations are highlighted. **(j)** Log2FC z-score of TSS single perturbations (top, IL2+/NEG) and residuals from additive model for bidirectional perturbations (bottom) for all TSS-E gRNA pairs with abs(log2FC) > 2 from a subsequent validation screen. **(a-i)** Data from *IL2* initial screen, n = 6 (3 biological replicates, 2 gRNAs per enhancer). **(j)** Data from *IL2* locus validation screen, n = 24 for grouped single perturbations and n = 147–168 for bidirectional perturbations (n = 3 biological replicates, 7–8 gRNAs per enhancer). **(i)** Significance cutoff FDR ≤ 0.05. **(j)** Boxplot, median and IQR. Box whiskers, 1.5x IQR. **(i,j)** Two-sided Wilcoxon test. Significance tested relative to (i) NTC, or (j) weakest TSS gRNA. Significance cutoffs: ns p > 0.05, * p ≤ 0.05, ** p ≤ 0.01, *** p ≤ 0.001, **** p ≤ 0.0001.

We designed a CRISPRai gRNA pool for REs of *IL2* and studied the effect of these perturbations on cytokine expression in human Jurkat T cells. Specifically, we designed CRISPRai gRNAs targeting 10 predicted enhancers and the promoter (Fig. 3a and Extended Data Fig. 5a–i). *IL2* is a key cytokine gene with a relatively large set of predicted enhancers, spanning a 2.4-Mb range^45^, providing an opportunity to study enhancer interactions in both short and long range (Fig. 3a and Extended Data Fig. 5f). We selected predicted enhancers with high enhancer scores for *IL2* in the ABC model^44,45^. Some selected enhancers exhibited strong enhancer-related epigenomic features, whereas others did not (Fig. 3a). In the gRNA pool, we included 576 gRNA pairs (484 bidirectional double, 88 single and four NTC gRNA pairs; Fig. 3b, Extended Data Fig. 2b and Supplementary Table 4). The gRNA pool contained all CRISPRa and CRISPRi single perturbations and all CRISPRai pairwise combinations for each enhancer and the TSS as well as NTCs (Extended Data Fig. 2b). We introduced the lentiviral pool of gRNAs to our CRISPRai-expressing Jurkat T cell line (Extended Data Fig. 1f–i). After 6 d of CRISPRai induction, the cells were activated to induce cytokine expression and sorted for cytokine positive and negative populations using both IL2 and IFNG expression (Fig. 3c, left, and Extended Data Fig. 2d); then, gRNA enrichment libraries were constructed (Fig. 3b and Extended Data Fig. 5a–e), and all CRISPRa and CRISPRi pairs were examined (Fig. 3d and Extended Data Fig. 5g–i).

**Extended Data Fig. 6:**
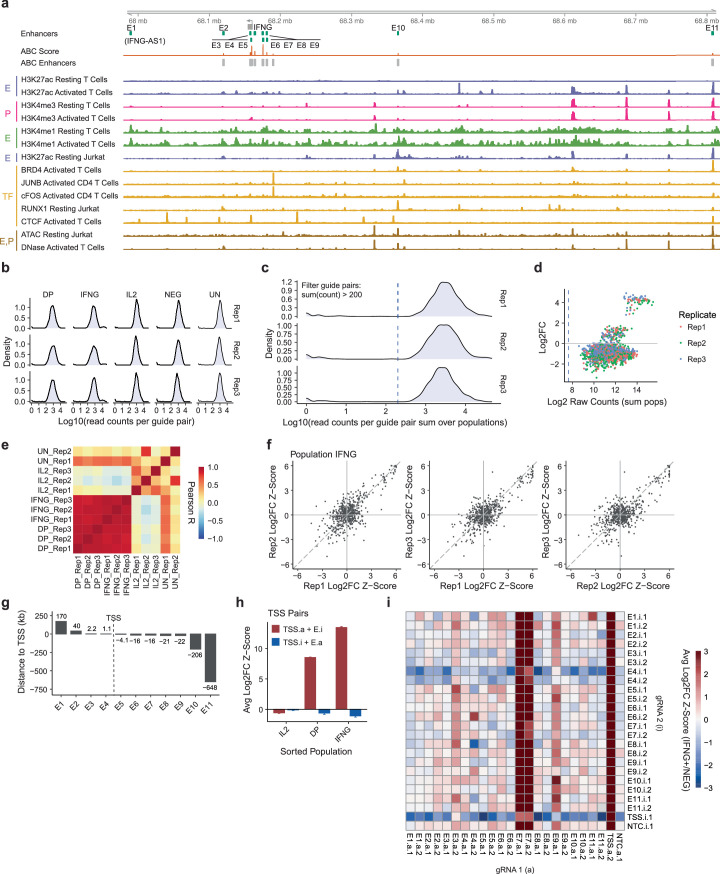
(related to Fig. 3). Additional data from Jurkat *IFNG* locus initial screen. **(a)** Genome tracks showing regulatory landscape of *IFNG* gene locus. **(b)** Log10 raw read counts per gRNA pair by sorted population and biological replicate. DP = IL2 + IFNG+ (double positive), IFNG = IL2-IFNG+, IL2 = IL2 + IFNG-, NEG = IL2-IFNG- (double negative), UN = unsorted. **(c)** Log10 raw read counts per gRNA pair summed over all populations, with read count cutoff (blue dotted line) used for filtering reads. **(d)** Log2FC (IFNG+/NEG) versus log2 raw read counts per gRNA, colored by biological replicate. **(e)** Pearson correlation of log2FC (IFNG+/NEG) across all gRNAs for sorted populations and biological replicates. **(f)** Log2FC z-score (IFNG+/NEG) correlation of biological replicates for IFNG sorted population. **(g)** Distance between enhancer midpoint and *IFNG* TSS**. (h)** Average log2FC z-score (IFNG+/NEG) for all single and bidirectional TSS-E pairs, binned by sorted population. Data are mean ± SEM**. (i)** Average log2FC z-score (IFNG+/NEG) for all single, bidirectional, and NTC gRNA pairs. Columns and rows indicate gRNA1 and gRNA2 in the pair. **(a-i)** Data from *IFNG* screen, n = 6 (3 biological replicates, 2 gRNAs per enhancer).

For further IL2 gRNA pool analysis, we focused on comparing IL2 single-positive cells relative to cytokine-negative cells (that is, IL2^+^ versus NEG) to investigate how *IL2* locus perturbations influence IL2 expression. In addition to the *IL2* locus gRNA pool, we also designed an *IFNG* locus gRNA pool with 625 bidirectional gRNA pairs targeting 11 predicted enhancers and the promoter at this locus (Fig. 3c, right, Extended Data Fig. 6a–i and Supplementary Table 5). For the IFNG gRNA pool analysis, we focused on comparing IFNG single-positive cells relative to cytokine-negative cells (that is, IFNG^+^ versus NEG) to investigate how *IFNG* locus perturbations influence IFNG expression. For most of our analysis, we calculated log_2_FC gRNA enrichment z-scores relative to NTC gRNAs, which we refer to as log_2_FC z-scores.

**Extended Data Fig. 7:**
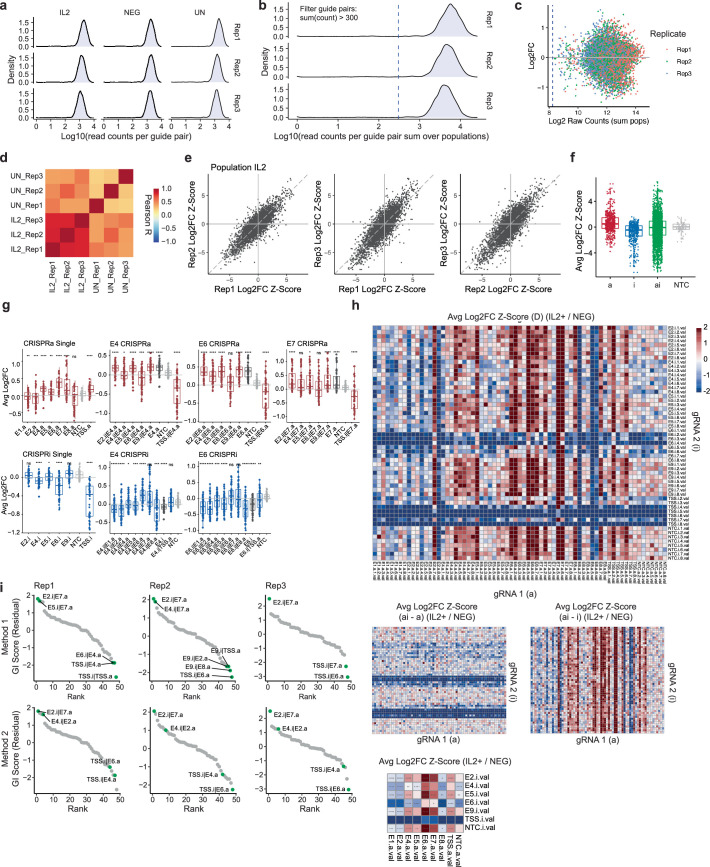
(related to Figs. 3–4, 6). Additional data from Jurkat *IL2* locus validation screen. **(a)** Log10 raw read counts per gRNA pair by sorted population and biological replicate. IL2 = IL2+, NEG = IL2-, UN=unsorted. **(b)** Log10 raw read counts per gRNA pair summed over all populations, with read count cutoff (blue dotted line) used for filtering reads. **(c)** Log2FC (IL2+/NEG) versus log2 raw read counts per gRNA, colored by biological replicate. **(d)** Pearson correlation of log2FC (IL2+/NEG) across all gRNAs for sorted populations and biological replicates. **(e)** Log2FC z-score (IL2+/I NEG) correlation of biological replicates for IL2 sorted population. **(f)** Average log2FC z-score (IL2+/NEG) for all single (a or i), bidirectional (ai), or NTC gRNAs. **(g)** log2FC (IL2+/NEG) of selected single and selected bidirectional perturbations. **(h)** Average log2FC z-score (IL2+/NEG) for all single, bidirectional, and NTC gRNA pairs as observed (top), and with CRISPRa or CRISPRi gRNA contribution subtracted (middle). Average log2FC z-score averaged over 8 gRNAs per enhancer (bottom). **(i)** Genetic interaction score analysis for all gRNA pairs, showing significance method 1: pairs >1 standard deviation from the mean of residuals and hit shared by all 3 replicates (top), and significance method 2: normal distribution p-value < 0.05 (bottom). **(a-i)** Data from *IL2* locus validation screen, n = 147–168 (n = 3 biological replicates, 7–8 gRNAs per enhancer). **(f,g)** Boxplot, median and IQR. Box whiskers, 1.5x IQR. **(g,h)** Two-sided Wilcoxon test, BH correction. Significance cutoffs: ns p > 0.05, * p <= 0.05, ** p <= 0.01, *** p <= 0.001, **** p <= 0.0001.

First, we investigated general trends in enhancer–promoter interactions. For the IL2 screen, we compared log_2_FC z-scores in IL2^+^ versus cytokine-negative populations (IL2^+^/NEG) and found that TSS–E interactions followed a largely additive relationship with respect to log_2_FC z-score (expected versus observed log_2_FC z-score: R^2^ = 0.91, *P* ≤ 2 × 10^−16^, slope = 0.97; Fig. 3e). log_2_FC z-scores ranged from approximately −20 to +7.5 (Fig. 3e), and log_2_FC ranged from −1.05 to +0.62 for CRISPRi and CRISPRa, respectively. For IFNG, log_2_FC z-scores ranged from approximately −3.5 to +14 (Fig. 3j). We noted that some *IL2* enhancers had strong functional effects, whereas others had weaker functional effects, in single perturbations (Fig. 3d). Additionally, we observed a trend that TSS–E bidirectional perturbations became less additive as TSS perturbation strength increased when considering pairs with an enhancer gRNA passing a threshold (abs(log_2_FC z-score) > 2) in a subsequent validation gRNA pool (Extended Data Fig. 5j), where we leveraged the natural variation in TSS gRNA strength by binning TSS–E bidirectional perturbations based on the corresponding TSS single perturbation strength. The distribution of residuals centered on zero for pairs with low TSS gRNA strength and shifted up for TSS.a and down for TSS.i pairs with greater TSS gRNA strength. Furthermore, in general, the TSS exhibited clear hierarchy over enhancers (Fig. 3g–i and Extended Data Figs. 5h, 6i and 7h). In other words, the TSS perturbation was functionally dominant over enhancer perturbations and, therefore, acted as the driver of target gene expression. Repressing the promoter prevented most of the activated enhancers from activating *IL2* or *IFNG* and vice versa (Fig. 3g,j).

Next, we investigated interactions between the promoter and each individual enhancer to uncover potential enhancer-specific effects. For *IL2*, two enhancers had strong functional effects that were capable of overcoming TSS perturbation, namely E4 and E6 (Fig. 3g). Repression of these two enhancers individually was sufficient to counteract TSS activation and significantly reduce target gene expression (Fig. 3g). In the reverse condition (E4.a|TSS.i and E6.a|TSS.i), both of these enhancers exhibited the ability to counteract TSS perturbation, as evidenced by both screens for E4.a|TSS.i and by the significant (*P* ≤ 1 × 10^−4^) and large effect size for E6.a|TSS.i relative to TSS.i observed in a subsequent validation screen where a larger number of gRNAs enabled us to observe this effect (Fig. 3h and Extended Data Fig. 7h). Together, this behavior suggests that E4 and E6 may act like ‘gatekeepers’ for *IL2* expression, in that they are strong functional enhancers that, when perturbed, are capable of strongly dimming the perturbation applied to the TSS. For *IFNG*, E4.i minimally counteracted TSS.a, and E7.a strongly counteracted TSS.i (Fig. 3j).

After identifying the existence of gatekeeper enhancers capable of counteracting TSS perturbation, we investigated these enhancers further. We designed a second gRNA pool to validate findings from the initial screen and investigate enhancer function over a broader genomic range. We selected a subset of enhancers from the initial *IL2* locus screen; designed eight additional gRNAs for each enhancer, including all E–E and TSS–E CRISPRai pairs as well as NTCs, for a pool of 4,032 gRNA pairs (3,072 bidirectional double, 896 single and 64 NTC gRNAs, made up of 56 unique CRISPRi and 72 unique CRISPRa gRNAs; Fig. 3h, top, Extended Data Fig. 7h and Supplementary Table 6); and constructed gRNA enrichment libraries for IL2^+^ and IL2^−^ (NEG) populations (Extended Data Fig. 7a–e). In the validation screen, log_2_FC z-scores ranged from approximately −5 to +7.5 (Extended Data Fig. 7e), and log_2_FC ranged from approximately −1.2 to +1.3, for CRISPRi and CRISPRa, respectively (Extended Data Fig. 7g). The validation screen confirmed the gatekeeper effects of E4 and E6 and highlighted the presence of a strong activating functional hotspot within E7 that was capable of overpowering TSS perturbation (Fig. 3h, bottom). When quantifying the strength of single perturbations for gatekeeper enhancers, E4, E6 and the E7 hotspot exhibited 99%, 115% and 160% of TSS CRISPRa strength, and E4 and E6 exhibited 16% and 45% of TSS CRISPRi strength (Fig. 3i and Extended Data Fig. 7h). Across bidirectional perturbations, we observed strong concordance between gRNAs for the same enhancer. Quantitatively, out of the eight validation gRNAs per enhancer 7/8, 7/8, 8/8 and 6/8 are strongly directionally concordant for E4.a, E6.a, E4.i and E6.i, respectively (Extended Data Figs. 5h and 7h). For E4.i and E6.i, both gRNAs from the initial screen were concordant with the validation screen majority (Extended Data Figs. 5h and 7h). For E4.a, E6.a and E7.a, at least one of two gRNAs from the initial screen was concordant with the validation screen majority (Extended Data Figs. 5h and 7h). Furthermore, E4 and E6 demonstrated gatekeeper behavior in reciprocal CRISPRai conditions (that is, ai and ia) (Fig. 3i, bottom). We noted that CRISPRa appears more focal than CRISPRi, possibly due to different mechanisms of chromatin remodeling induced by VPR and KRAB (Extended Data Figs. 5h and 7h).

To confirm that off-target effects did not play a major role in our results, we performed a genome-wide analysis of potential off-target sites (Supplementary Table 7). We overlapped all putative gRNA off-target sites with the CRISPRa and CRISPRi screening data from previously published screens studying IL2 and IFNG^50^. Overall, 0.07% (14/19,999) of off-target sites overlapped a gene that may be involved in IL2 or IFNG regulation; 6.3% (13/204) of gRNAs had at least one off-target site at one of these genes; and most of these off-target sites had four mismatches. It has been shown that two mismatches typically render a gRNA non-functional for CRISPRko^78^ and CRISPRi^79^. Thus, off-target overlap with coding genes is unlikely to play a major role in our results.

### CRISPRai defines enhancer–enhancer regulatory interactions

**Fig. 4:**
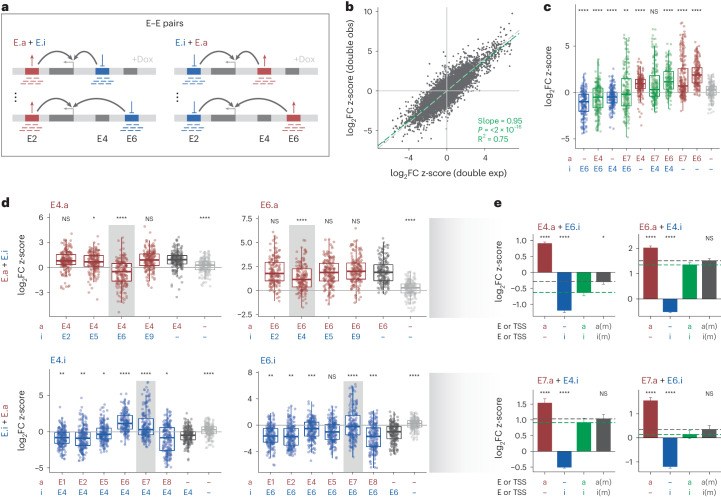
CRISPRai reveals hierarchies in enhancer–enhancer interactions for IL2 transcriptional regulation. **a**, Schematic of bidirectional E–E perturbation pairs in *IL2* locus validation screen. **b**, log_2_FC z-score (IL2^+^/NEG) for all E–E pairs, showing expected and observed. Expected log_2_FC z-score was calculated using additive model of single perturbations. **c**, log_2_FC z-score (IL2^+^/NEG) for selected single and bidirectional perturbations for E–E pairs showing tuning of *IL2* expression. **d**, log_2_FC z-score (IL2^+^/NEG) for all E–E pairs containing E4 (left) and E6 (right) for CRISPRa (top) or CRISPRi (bottom) of E4 and E6, respectively. Gray bars highlight gatekeeper enhancer perturbation pairs, which are shown further in **e**. **e**, Examples of CRISPRai for specific E–E pairs containing E4, E6 and E7. Bins represent single (a or i), bidirectional (ai) and expected bidirectional perturbation from additive model (ai model, gray); dashed lines show observed and expected bidirectional perturbations. Data are mean ± s.e.m. **a**–**e**, Data from *IL2* locus validation screen. **c**–**e**, *n* = 168–192 (three biological replicates, 7–8 gRNAs per enhancer, includes gRNAs for entire E7 region including hotspot). **b**, Linear regression. **c**,**d**, Box plot, median and interquartile range (IQR). Box whiskers, 1.5× IQR. **c**–**e**, Two-sided Wilcoxon test. Significance was tested relative to single perturbation of indicated enhancer (**d**) and observed bidirectional perturbation (**e**). Significance cutoffs: NS *P* > 0.05, *P ≤ 0.05, **P ≤ 0.01, ***P ≤ 0.001, ****P ≤ 0.0001. exp, expected; NS, not significant; obs, observed.

We next investigated how enhancers interact with other enhancers to control gene regulation. We compared the log_2_FC z-scores of E–E bidirectional perturbations from the *IL2* locus validation screen (Fig. 4a). Similar to the TSS–E pairs, E–E pairs largely followed an additive model with respect to log_2_FC z-score (R^2^ = 0.75, *P* ≤ 2 × 10^−16^, slope = 0.95; Fig. 4b). Single and bidirectional E–E perturbations enabled tuning of *IL2* expression over a broad range, supporting a hypothesis that multiple enhancers of varying strengths enable more precise tuning collectively than would be possible with fewer enhancers (Fig. 4c). Notably, the gatekeeper enhancers identified from the TSS–E bidirectional perturbations, E4, E6 and E7, showed similar gatekeeper behavior when paired with other enhancers (Fig. 4d and Extended Data Fig. 7g). E4 or E6 activation increased gene expression when other enhancers in the same locus were repressed, and, conversely, E4 or E6 repression prevented gene expression even if other IL2 enhancers were activated.

To investigate the outcome of perturbing two gatekeeper enhancers simultaneously, we further examined the interactions among E4, E6 and E7. E6 repression counteracted E4 activation (Fig. 4d, top left), and, conversely, E6 activation counteracted E4 repression (Fig. 4d, bottom left). We observed similar trends in magnitude of enhancer strength as seen for TSS–E pairs (Fig. 3g,h), supporting the strong and moderate functional effects of E6 and E4, respectively. E7 activation was also capable of counteracting E4 and E6 repression (Fig. 4d, bottom). All other enhancers had minimal ability to counteract E4 and E6 perturbation (Fig. 4d). Interestingly, E1 and E2 activation weakly reduced log_2_FC z-score, suggesting that these two enhancers, which are both approximately 1.2 Mb from the TSS, may be weak repressive REs (Fig. 4d and Extended Data Fig. 7h). Additionally, CRISPRai of E4 and E6 enabled reversible control of *IL2* expression (Fig. 4e, top). Furthermore, the relationship between E4 and E6 was additive or nearly additive regardless of the perturbation direction (ai versus ia) (Fig. 4e, top). E7 activation counteracted repression of both E4 and E6, and these relationships were additive (Fig. 4e, bottom).

### IL2 enhancer activity in primary human and chimeric antigen receptor T cells

**Fig. 5:**
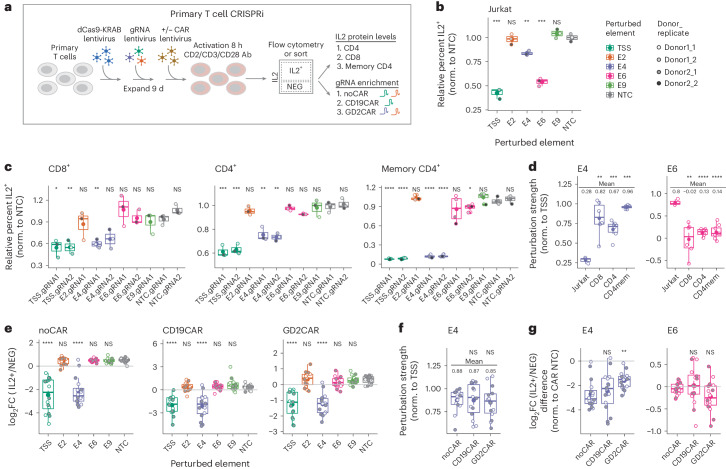
IL2 enhancer activity in primary human T cells and CAR T cells. **a**, Schematic of primary T cell CRISPRi, including a summary of cell subtypes and CAR T cell conditions as well as the readouts used. **b**, Intracellular cytokine staining by flow cytometry during enhancer CRISPRi in Jurkat T cells. *n* = 3 (three biological replicates). **c**, Intracellular cytokine staining by flow cytometry during enhancer CRISPRi in human primary T cells (CD8^+^, CD4^+^ and memory CD4^+^). CD8^+^ and CD4^+^ cells are gated from bulk CD3^+^ T cells, and memory CD4^+^ cells were isolated before ex vivo culture via bead-based enrichment. *n* = 3 (three donors) for CD4^*+*^ and CD8^+^ T cells and *n* = 4 (two donors, two technical replicates) for CD4^+^ memory T cells. **d**, Enhancer perturbation strength, normalized to TSS perturbation for data from **a** and **c**. **e**, CRISPRi gRNA enrichment screen in human memory CD4^+^ primary T cells for non-CAR, CD19-28z-CAR and HA-GD2–28z-CAR cells. *n* = 16 (two donors, eight gRNAs per enhancer). log_2_FC (IL2^+^/NEG) is shown for each CAR condition. **f**, Enhancer perturbation strength, log_2_FC (IL2^+^/NEG) normalized to TSS perturbation. **g**, log_2_FC (IL2^+^/NEG) normalized to NTC cells. Number of gRNAs per enhancer: two (**b**–**d**) and eight (**e**–**g**). **b**–**g**, Box plot, median and interquartile range (IQR). Box whiskers, 1.5× IQR. **b**–**d**, Two-sided *t*-test. **e**–**g**, Two-sided Wilcoxon test. Significance was tested relative to indicated group. Significance cutoffs: NS *P* > 0.05, *P ≤ 0.05, **P ≤ 0.01, ***P ≤ 0.001, ****P ≤ 0.0001. NEG, negative; norm., normalized; NS, not significant.

We next extended our findings from CRISPRai to several primary cell contexts. We performed individual and pooled CRISPRi perturbations in primary human T cells and chimeric antigen receptor (CAR) T cells. We included gRNAs for gatekeeper enhancers (E4 and E6), the TSS, the NTC and negative control enhancers that exhibited minimal effect on IL2 expression in the Jurkat screens (E2 and E9), and we followed a similar experimental workflow as the Jurkat IL2 gRNA enrichment screens (Fig. 5a). First, we individually validated selected enhancer CRISPRi perturbations and quantified enhancer perturbation strength during CRISPRi in Jurkat T cells using flow cytometry for intracellular IL2 (Fig. 5b). We observed similar trends in enhancer strength as seen in the Jurkat gRNA enrichment screens, thus validating the gatekeeper effects of these enhancers (Fig. 5b). Next, we performed individual CRISPRi (*ZIM3* KRAB domain) perturbations in primary human T cells, including bulk CD3^+^ cells (gated for CD4^+^ and CD8^+^) and isolated CD4^+^ memory cells. We prioritized the memory CD4^+^ T cell population for in-depth study because Jurkat cells are CD4^+^ and because previously published assay for transposase-accessible chromatin with sequencing (ATAC-seq) data showed that, among primary T cell subsets, CD4^+^ memory T cells have the highest accessibility at E4, E6 and the *IL2* TSS^80^. We found that E4 had the greatest effect among enhancers in primary T cells when repressed (Fig. 5c). Furthermore, we noted that there is likely greater context-dependent usage of enhancers in primary T cells relative to Jurkat T cells; a subtle effect was observed for E6 in isolated CD4^+^ memory T cells with one gRNA, suggesting that E6 likely has context-restricted function in primary cells (Fig. 5c). Quantitatively, E4 perturbation strength varied across T cell subsets; on average, E4 achieved 28%, 82%, 67% and 96% of TSS perturbation strength for Jurkat, CD8^+^, CD4^+^ and CD4^+^ memory primary T cells, respectively (Fig. 5d, left). On average, E6 achieved 80% and 14% of TSS perturbation strength for Jurkat and CD4^+^ memory primary T cells, respectively (Fig. 5d, right).

**Extended Data Fig. 8:**
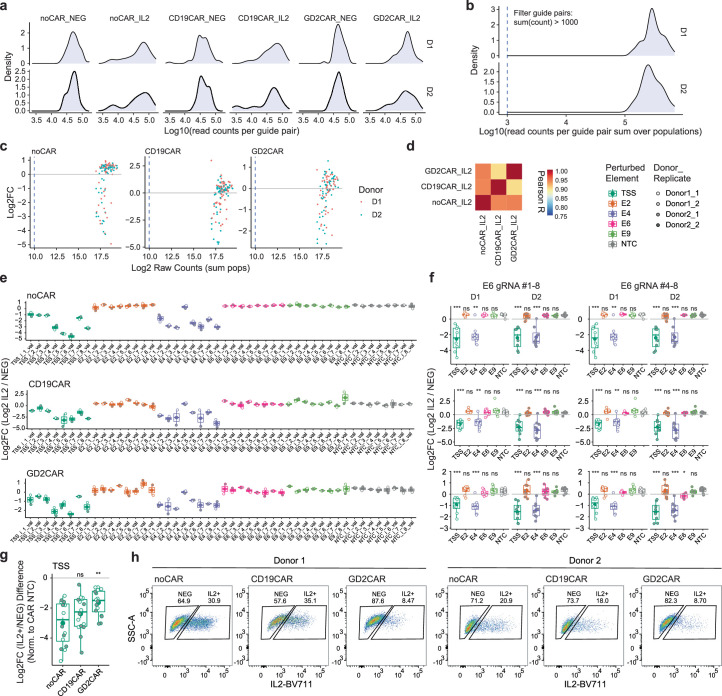
(related to Fig. 5). Additional data from primary T cell *IL2* locus screens. **(a)** Log10 raw read counts per gRNA pair by sorted population and biological replicate. IL2 = IL2 + , NEG = IL2-. **(b)** Log10 raw read counts per gRNA pair summed over all populations, with read count cutoff (blue dotted line) used for filtering reads. **(c)** Log2FC versus log2 raw read counts per gRNA, colored by biological replicate. **(d)** Pearson correlation of log2FC (IL2/NEG) across all gRNAs for sorted populations and biological replicates. **(e)** Log2FC (IL2+/NEG) for all gRNAs in primary human T cells with no CAR, CD19-28z-CAR, and HA-GD2–28z-CAR. **(f)** Same data as (e), binned by RE, for all 8 gRNAs aggregated (left), or only E6 gRNAs 4-8 aggregated (right). **(g)** Log2FC (IL2+/NEG) normalized to NTC cells. **(h)** Intracellular IL2 by flow cytometry. **(a-f)** Data from 2 biological replicates (2 donors) in human primary memory CD4+ cells. **(f,g)** n = 16 (2 donors, 8 gRNAs per enhancer). **(e-g)** Boxplot, median and IQR. Box whiskers, 1.5x IQR. **(f,g)** Two-sided Wilcoxon test. Significance tested relative to NTC. Significance cutoffs: ns p > 0.05, * p ≤ 0.05, ** p ≤ 0.01, *** p ≤ 0.001, **** p ≤ 0.0001.

We next performed pooled CRISPRi screens in both CD19-28z (clinically approved) and HA-GD2-28z (exhaustion prone^81,82^) CD4^+^ memory primary CAR T cells (Fig. 5a,e–h and Extended Data Fig. 8a–h). We observed similar trends in enhancer perturbation effects in CAR T cells as for non-CAR primary T cells (Fig. 5e and Extended Data Fig. 8e,f). We found that E4 perturbation strength relative to TSS perturbation strength was not influenced by level of CAR T cell exhaustion state. On average, E4 exhibited 88%, 87% and 85% of TSS perturbation strength for non-CAR, CD19-28z-CAR and HA-GD2-28z-CAR T cells, respectively (Fig. 5f). In contrast, CAR T cell capacity for perturbation by E4 varied with degree of CAR T cell activation or exhaustion state; magnitude of E4 perturbation was largest in non-CAR T cells and grew progressively smaller for CD19-28z and HA-GD2-28z CAR T cells (Fig. 5g, left). This trend was also observed for the TSS (Extended Data Fig. 8g). E6 perturbation in CAR T cells was detected only when considering gRNAs in the 5′ end of E6 (Extended Data Fig. 8f). However, after correcting log_2_FC for variability in NTC gRNAs, we observed that CAR T cell capacity for E6 perturbation was slightly increased in HA-GD2-28z-CAR T cells (Fig. 5g, right). Additionally, HA-GD2-28z-CAR T cells had less than half the amount of IL2^+^ cells compared to non-CAR and CD19-28z-CAR T cells (Extended Data Fig. 8h), indicating a reduced capacity for IL2 production in HA-GD2-28z-CAR T cells, as expected in T cell exhaustion^81,82^.

### Epigenomic analysis reveals activity-driven and contact-driven REs

**Fig. 6:**
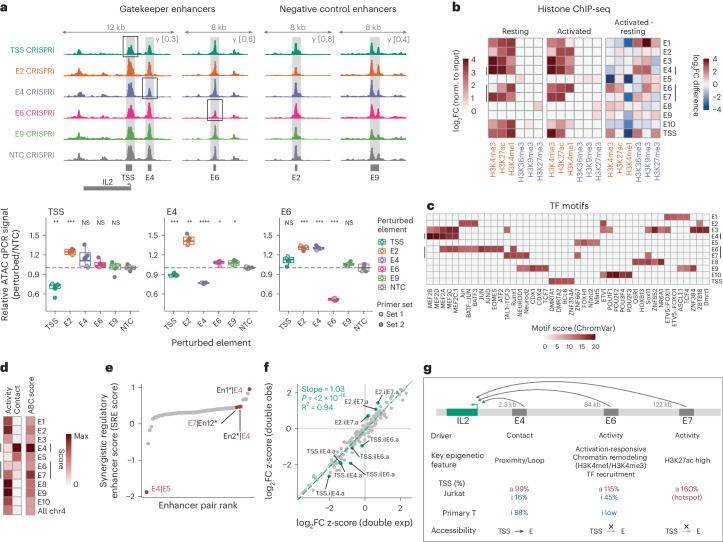
Epigenomic interrogation of CRISPRai enhancers reveals activity-driven and contact-driven REs in the *IL2* locus. **a**, ATAC-seq profiles of Jurkat T cells with CRISPRi perturbation of the indicated RE, shown as genome tracks (top) and quantification from ATAC–qPCR for peaks overlapping REs. Black boxes indicate the peak for the CRISPRi-targeted RE. *n* = 2 (two biological replicates, top) and *n* = 4 (two qPCR technical replicates, two primer pairs, bottom). **b**, ENCODE histone ChIP-seq^70,71^ from resting and activated primary T cells, with activating marks (orange) and repressive marks (purple). **c**, Motif scores (ChromVar^97^) for JASPAR TF motifs^85^ present in each enhancer. **d**, Activity, contact and ABC scores from the ABC model^45^. **e**, SRE scores for all E–E pairs in the SRE model^30^. Selected enhancer pairs that also contain enhancers from the CRISPRai screen are annotated, and CRISPRai enhancers are highlighted in red. Each dot is a pair. **f**, log_2_FC z-score (IL2^+^/NEG) for bidirectional perturbations, showing expected and observed. Expected log_2_FC z-score was calculated using additive model of single perturbations. Pairs with significant genetic interaction scores are highlighted (green; significance cutoffs are described in the Methods). Each dot is an enhancer pair from one biological replicate. **g**, Proposed model of enhancer-mediated gene regulation for *IL2* by strong functional enhancers. **f**, Data from *IL2* locus validation screen, *n* = 3 (three biological replicates, 7–8 gRNAs per enhancer are aggregated). **a**, Box plot, median and interquartile range (IQR). Box whiskers, 1.5× IQR. Two-sided *t*-test. Significance was tested relative to NTC. **f**, Linear regression. Significance cutoffs: NS *P* > 0.05, *P ≤ 0.05, **P ≤ 0.01, ***P ≤ 0.001, ****P ≤ 0.0001. exp, expanded; norm., normalized; NS, not significant; obs, observed.

**Extended Data Fig. 9:**
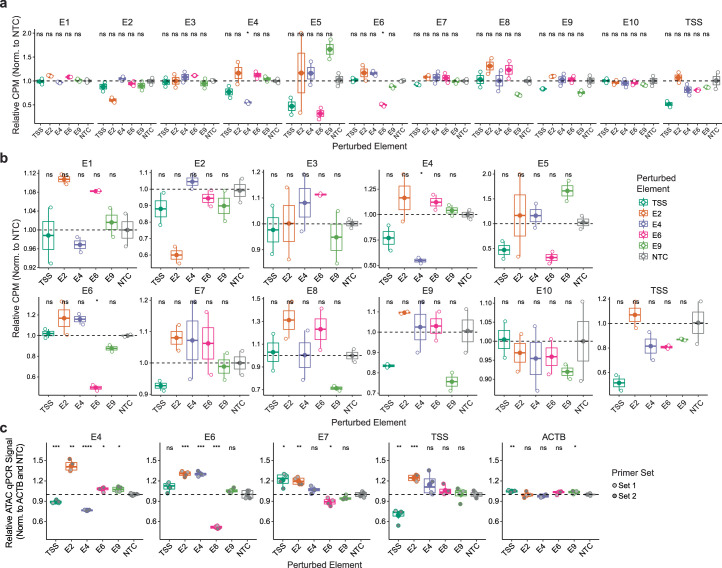
(related to Fig. 6). Chromatin accessibility at IL2 enhancers during enhancer CRISPRi in Jurkat. **(a)** ATAC-seq at all enhancers and the TSS during CRISPRi in Jurkat T cells, with same y axis. (**b)** Same as (a) for different y axis. **(c)** ATAC-qPCR for peaks overlapping enhancers, the TSS, and negative control *ACTB* peaks. **(a,b)** n = 2 (2 biological replicates). **(c)** n = 4 (2 qPCR technical replicates, 2 primer pairs). **(a-c)** Boxplot, median and IQR. Box whiskers, 1.5x IQR. Two-sided t-test. Significance tested relative to NTC. Significance cutoffs: ns p > 0.05, * p ≤ 0.05, ** p ≤ 0.01, *** p ≤ 0.001, **** p ≤ 0.0001.

In addition to the gRNA enrichment and intracellular protein data demonstrating gatekeeper enhancer effects, we sought to further validate IL2 enhancers and dissect the mechanism underlying gatekeeper enhancer-mediated *IL2* gene regulation. To provide mechanistic insight, we performed ATAC-seq on RE perturbed cells (Fig. 6a and Extended Data Fig. 9a–c) and integrated these data together with previously published ChIP-seq (ENCODE^70,71^) and ABC model^44,45^ datasets to jointly assess chromatin accessibility, histone modifications, TF motif enrichment and chromatin looping (Fig. 6b–d).

We first assessed chromatin accessibility changes induced by CRISPRi perturbation of selected enhancers using the same set of enhancers as the primary T cell experiments. We performed ATAC-seq on perturbed Jurkat T cells, as well as ATAC–qPCR, which quantitatively detects accessibility changes at specific loci of interest^83^. We observed that repressing one enhancer by CRISPRi significantly decreased chromatin accessibility at that enhancer (Fig. 6a). In all conditions, the perturbed RE had the greatest decrease in accessibility when considering all IL2 REs but had a limited ability to affect accessibility at distant enhancers (Fig. 6a and Extended Data Fig. 9a–c). For example, E4 repression did not alter accessibility of E2 or E7, and E6 repression minimally altered accessibility of E4 and E7. Additionally, gatekeeper enhancer function could not be completely explained by their impact on promoter accessibility. CRISPRi of enhancers E4 and E6 did not reduce TSS accessibility despite resulting in IL2 protein reduction as measured by flow cytometry (reduced to 71% and 33% of NTC level, respectively; Fig. 5b). This result indicates that enhancer-mediated induction of IL2 expression is not entirely driven by chromatin accessibility; rather, these enhancers likely function through other biochemical means, such as RNA polymerase II pause release, TF recruitment or histone modification spreading.

**Extended Data Fig. 10:**
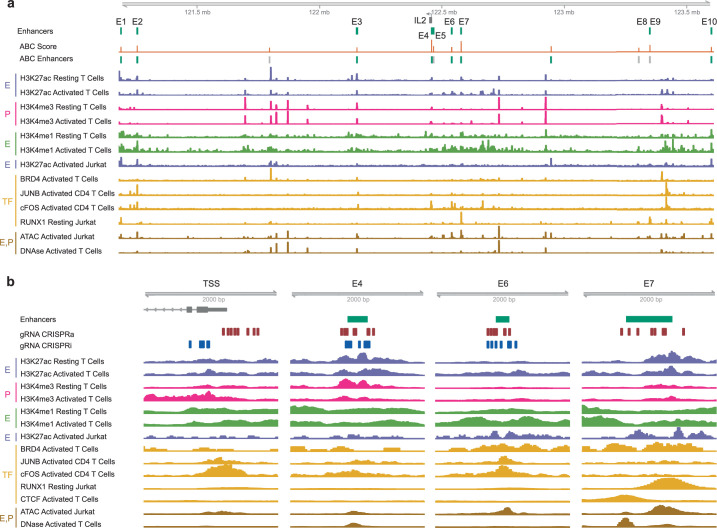
(related to Figs. 3–6). Genome tracks for strong functional enhancers of *IL2.* **(a)** Genome tracks showing regulatory and epigenetic landscape across *IL2* gene locus. **(b)** Genome tracks at selected enhancers and the TSS.

To investigate these possibilities, we compared ENCODE histone ChIP-seq in resting and activated primary T cells^70,71^ (Fig. 6b and Extended Data Fig. 10a,b), a relevant comparison because our screen endpoint was T cell activation (Fig. 3b). We found that E4 and E7 had high to moderate activating histone marks, including H3K4me3 and H3K27ac (Fig. 6b and Extended Data Fig. 10b). In contrast, E6 was relatively low for these histone marks but showed a large increase in activating histone marks in activated compared to resting cells. The most prominent histone mark for E6 was H3K4me1, which was accompanied by H3K4me3 depletion, a characteristic of primed enhancers^84^ (Fig. 6b and Extended Data Fig. 10b). In addition, compared to other enhancers, E6 was highly enriched for TF motifs (JASPAR)^85^ involved in T cell activation, including BATF3, JUN, JUND, ATF2 and EOMES, indicating that E6 is activation responsive and suggesting that it may be important for regulating activation-induced IL2 expression in T cells (Fig. 6c). TF ChIP-seq in activated primary CD4^+^ T cells corroborated AP-1 family TF occupancy at E6 (Extended Data Fig. 10b)^86^. Together, these findings suggest that E6 is a primed enhancer in primary T cells; however, its heightened ability to recruit TFs gives it the potential to be highly activation responsive, which may contribute to its context-restricted function in primary T cells and strong gatekeeper function in Jurkat T cells.

Additionally, we leveraged the ABC model^44,45^ data to investigate further epigenomic characteristics of IL2 enhancers. Under the ABC model, E4 had the highest predicted enhancer score, with high contact score (contact frequency with the TSS) yet low activity score (combined score of epigenetic features)^45^ (Fig. 6d). Thus, E4 gatekeeper function is likely primarily contact driven rather than activity driven. Conversely, E7 exhibited the opposite, with low contact score but high activity score, resulting in a relatively high overall predicted enhancer score, suggesting activity-driven function (Fig. 6d). E6 had intermediate scores for both contact and activity (Fig. 6d). Taken together, these attributes indicate that E–TSS contacts and enhancer activity likely represent complementary mechanisms, where either property is able to drive enhancer-mediated gene regulation in a context-specific manner.

To quantify the extent of genetic interactions among IL2 REs, we sought to contextualize our results using previously published approaches for studying genetic interactions^30,87^. First, we investigated whether any strong functional CRISPRai enhancers overlapped with the splicing regulatory element (SRE) enhancer set identified in Lin et al.^30^. We found that E7 and, most notably, E4 were present in the top most synergistic SRE E–E pairs, confirming their importance in *IL2* gene regulation (Fig. 6e). E6 was not present in the SRE enhancer set. Second, we calculated ‘GI scores’, using a method similar to Horlbeck et al.^87^. We defined GI scores as the residual between the linear model and the observed bidirectional perturbation log_2_FC z-score. The resulting hits for synergistic interactions were E2.i|E7.a, TSS.i|E4.a and TSS.i|E6.a (positive residuals), whereas TSS.i|E4.a and TSS.i|E6.a were identified as buffering interactions (negative residuals) (Fig. 6f). In other words, E2.i|E7.a resulted in higher IL2 expression than expected, and TSS.i|E4.a and TSS.i|E6.a resulted in lower IL2 expression than expected (Fig. 6f and Extended Data Fig. 7i). This analysis highlighted three key insights. First, this analysis underscored the hierarchy that the promoter has over enhancers in governing gene expression. Second, the promoter genetic interaction effect was unique to CRISPRi, and we did not observe this interaction for the reciprocal TSS.a pairs, suggesting that E–TSS interaction is directionally dependent for *IL2*. Third, we observed a genetic interaction for E2.i|E7.a where IL2 expression was greater than expected. Interestingly, we also noted that E2 CRISPRi resulted in increased accessibility at the TSS and all gatekeeper enhancers E4, E6 and E7 (Extended Data Fig. 9c). Furthermore, although the magnitude of E4 and E6 accessibility change during E2 CRISPRi was similar to that achieved by TSS CRISPRi, only E7 demonstrated equivalent magnitude accessibility change in both of these conditions, suggesting a unique relationship between E2 and E7. Furthermore, in the IL2 validation screen, we observed that E2.a weakly reduces IL2 expression (Extended Data Fig. 7g,h), suggesting that E2 is a weak repressive element.

In summary, our integrated analyses revealed two main modes of gene regulation by gatekeeper enhancers: activity driven and contact driven. Contact-driven enhancers, such as E4, exhibited strong three-dimensional contacts with the TSS (Fig. 6d), and repression of either this enhancer itself or the TSS reduced accessibility of the enhancer (Fig. 6a and Extended Data Fig. 9a–c). In contrast, activity-driven enhancers, such as E6, did not form loops as strongly and did not exhibit reduced accessibility during TSS repression. Furthermore, although most of the RE pairs exhibited additive function, which is expected given that strong genetic interactions are rare^50^, CRISPRai enabled identification of three genetic interactions among IL2 REs (Fig. 6f). We synthesized these findings into a proposed model of *IL2* gene regulation (Fig. 6g).

## Discussion

We developed a bidirectional epigenetic editing system, called CRISPRai, to expand the toolkit for investigating genetic interactions and non-coding genetic elements. Furthermore, we extended CRISPRai to be compatible with single-cell readouts and demonstrated the utility of the system in applying bidirectional epigenetic perturbations to pairs of genes. This allowed us to uncover insights into the genetic interaction between SPI1 and GATA1, including that the bidirectional perturbation uniquely highlights synergistically regulated downstream target genes and that the pattern of SPI1 and GATA1 occupancy at downstream target genes depends on regulatory mode. Moving forward, future approaches could extend CRISPRai Perturb-seq to incorporate multi-omic readouts or to study non-coding disease-associated variants. Additionally, emerging technologies, such as cell hashing^88^; alternative single-cell workflows, such as split-pool^89^; and new lower-cost sequencing technologies^90^ are expanding the number of cells feasible to sequence per experiment and provide a clear path toward enhancing the scale of CRISPRai screens in the future, potentially toward extending genome-wide Perturb-seq^91^ for use with CRISPRai.

We also demonstrate here the utility of CRISPRai in studying non-coding elements. We applied CRISPRai to study hierarchies in gene regulation between the promoter and enhancers of *IL2* and extended our findings to primary T cells and CAR T cells. Integrated analysis of CRISPRai functional data with epigenomic datasets revealed the existence of gatekeeper enhancers, which exhibited strong functional effects capable of heavily competing with the promoter in regulating IL2 expression, and elucidated mechanisms of gatekeeper enhancer function. We anticipate that future applications of CRISPRai can further extend its capabilities for studying non-coding elements by multiplexing more than two simultaneous perturbations or using additional epigenetic effector domains, such as DNA methyltransferase or demethylase^92^. This will enable large-scale, systematic dissection of non-coding disease-associated variants.

New tools to manipulate coding and non-coding elements of the genome are needed to enable dissection of the complex gene regulatory and genetic interaction networks that wire mammalian cells. CRISPRai enables precise and bidirectional control over genes and REs in human cells, facilitating investigation of these questions. Specifically, CRISPRai revealed insight on the SPI1 and GATA1 hematopoietic lineage TFs. CRISPRai enabled modulation of erythroid and myeloid gene signatures using bidirectional perturbations as well as identification and quantification different modes of regulation on downstream target genes, highlighting its utility in mapping genetic networks. Additionally, CRISPRai can elucidate RE landscapes and enhancer mechanisms. It is known that enhancer functionality is heterogenous and complex; some enhancers act in an additive manner^76^, whereas other rare enhancers may have synergistic effects in combination^30^. Some enhancers offer redundancy, whereas others are dominant levers for gene expression control^76,77,93,94^. Enhancers differ in their structural chromatin contacts^95^, E–TSS distance^40^ and chromatin modifications^42^ and in which TFs they are capable of recruiting^46,47^, which likely governs their function and the target genes for which they are compatible. These characteristics of enhancers are consistent with our findings from CRISPRai examining over 4,000 enhancer perturbation pairs. We show that combined enhancer function is primarily additive and that multiple enhancers enable tuning of gene expression levels. Furthermore, our ability to perform bidirectional perturbations revealed the existence of dominant gatekeeper enhancers that exist and heavily compete with the promoter. Additionally, Brosh et al.^96^ recently performed *Sox2* enhancer genome editing using long DNA assembly and sequence insertion in mouse embryonic stem cells, and they reported similar conclusions about enhancer hierarchies to those demonstrated by CRISPRai, which supports the biological significance of CRISPRai findings by corroborating the results with alternate methods for studying REs. Furthermore, Brosh et al. reported context-dependent function of REs within their gene locus, highlighting the importance of studying REs in their endogenous locus, which is a strength of CRISPRai. In summary, we developed CRISPRai and applied this method to study the SPI1–GATA1 genetic interaction as well as IL2 regulatory hierarchies. We anticipate that future applications of CRISPRai will enhance understanding of the multifaceted and heterogenous mechanisms underlying genetic interactions and gene regulation across the genome.

## Methods

### Cell culture of cell lines

Lenti-X HEK293T (Clontech) cells were cultured in DMEM (Gibco) with L-glutamine and sodium pyruvate supplemented with 10% FBS (Gibco) and 1% penicillin–streptomycin (Gibco) and passaged using TrypLE Express (Gibco). K562 (American Type Culture Collection (ATCC), CCL-238) was cultured in RPMI 1640 (Gibco) with L-glutamine supplemented with 10% FBS and 1% penicillin–streptomycin. Jurkat clone E6-1 cells (ATCC, TIB-152) were cultured in RPMI 1640 with L-glutamine (Gibco) supplemented with 10% FBS, 10 mM HEPES (Gibco), 1 mM sodium pyruvate (Gibco) and 1% penicillin–streptomycin. Cells were routinely tested for mycoplasma using a MycoAlert PLUS Detection Kit (Lonza) and found to be negative.

### Isolation and culture of primary human T cells

Human T cells were sourced from peripheral blood mononuclear cell (PBMC)-enriched leukapheresis products (Leukopaks, STEMCELL Technologies) from healthy donors, after institutional review board (IRB)-approved informed written consent (STEMCELL Technologies). T cell populations (bulk or CD4^+^ memory cells) were isolated from Leukopaks using EasySep magnetic selection following the manufacturer’s recommended protocol (STEMCELL Technologies, 100-0695, 19157). T cells were cultured in X-VIVO 15 (Lonza) supplemented with 5% FBS and 100 IU ml^−1^ recombinant human IL-2 (AmerisourceBergen).

### CRISPRai construct generation

The CRISPRai construct was cloned in the following format: TRE3G-VPR-dSaCas9-P2A-dSpCas9-BFP-KRAB-EF1a-Bleo-T2A-rtTA. The vector containing the TRE3G and Tet-On system was PiggyBac; the zeocin resistance gene and the Tet-On 3G transactivator were driven by the EF1a promoter (gifted by the Stanley Qi laboratory)^98^. The Super PiggyBac transposase plasmid was obtained from System Biosciences. VPR was obtained from pSLQ2349 (gifted by the Stanley Qi laboratory); dSaCas9 was obtained from pSLQ2840 (Addgene, 84246); and dSpCas9-BFP-KRAB was obtained from pHR-SFFV-dCas9-BFP-KRAB (Addgene, 46911). The *ZNF10* (*KOX1*) KRAB domain^7^ was used. Constructs were cloned using Gibson Assembly (NEBuilder HiFi DNA Assembly) and confirmed by Sanger sequencing (Elim Biopharmaceuticals). Primers and oligos were obtained from Elim Biopharmaceuticals and Integrated DNA Technologies (IDT). Selected constructs are available on Addgene (https://www.addgene.org/Howard_Chang/).

### CRISPR gRNA cloning

Primers and oligos for bulk validation experiments were obtained from Elim Biopharmaceuticals and IDT. Plasmids were confirmed by Sanger sequencing (Elim Biopharmaceuticals). Individual single gRNAs were cloned using Gibson Assembly (NEBuilder HiFi DNA Assembly). For validation and Perturb-seq experiments, gRNAs were constructed from pSLQ2853-3 pHR: U6-Sasgv2CXCR4-1 CMV-EGFP (Addgene, 84254) and pSLQ1852-2 pHR: U6-SpsgCD95-1 CMV-EGFP (Addgene, 84151). For dSaCas9 gRNAs, GFP was replaced with mScarlet (pmScarlet_Giantin_C1; Addgene, 85048).

For Perturb-seq single gRNAs, gRNAs pools were constructed from two gRNA backbones, with the dSpCas9 or dSaCas9 gRNA scaffold. Pools were cloned in arrayed format by ordering top and bottom approximately 31–33-bp gRNA oligos from IDT with appropriate overhangs. Top and bottom oligos were combined at 100 mM in annealing buffer (potassium acetate, 30 mM HEPES-KOH pH 7.4 and 2 mM magnesium acetate in water, adapted from Jonathan Weismann laboratory protocols, https://weissman.wi.mit.edu/resources/), annealed on a thermocycler at 95 °C for 4 min, cooled slowly for 3 h, pooled, phosphorylated using T4 PNK (NEB) at 37 °C for 30 min with 65 °C for 20-min PNK inactivation, ligated into the previously digested and dephosphorylated (Fast AP, Thermo Fisher Scientific) lentiviral gRNA backbone using T4 ligase (NEB) and transformed by heat shock into Stbl3 competent cells (Thermo Fisher Scientific).

For Perturb-seq double gRNAs, gRNA pools were constructed in a two-step cloning process (Extended Data Fig. 2a). Oligo pools (IDT) containing approximately 200-bp oligos were cloned with the format: (amplification primer)-(digest site)-(gRNA1)-(scaffold1)-(hu6 landing pad)-(digest site)-(amplification primer). For step 1, oligo pools were PCR amplified in multiple reactions with low cycle number (NEB Ultra II Master Mix), digested and size selected via gel purification (E-Gel EX, Thermo Fisher Scientific), ligated into predigested gRNA backbones with T4 ligase overnight at 16 °C for 16 h and inactivated at 65 °C for 10 min and transformed into Stbl3 competent cells and grown at 30 °C. For step 2, plasmid products were digested, dephosphorylated and gel size selected, and the previously digested hu6 PCR fragment (from pMJ117; Addgene, 85997) with appropriate overhangs was inserted via T4 ligation. Original vector backbone and intermediate backbone product were designed for digestion with Esp3I (BsmBI, NEB), and inserts were designed for digestion with BsaI (NEB).

For the enhancer gRNA enrichment screen, double gRNA pools were constructed in a one-step cloning process (Extended Data Fig. 2b). Primer pools were obtained from IDT and contained gRNA sequences and primer sequences for dSpCas9 gRNA scaffold and the hu6 promoter. Primers were used to generate a PCR product in the format of [mu6 fragment-gRNA1-Sp gRNA scaffold-hu6-gRNA2-Sa gRNA scaffold fragment], flanked by BsmBI digestion sites. The PCR product and backbone were digested separately and ligated with T4 ligase following recommended protocols.

gRNAs are listed in Supplementary Tables 4 and 7 for Jurkat flow cytometry and ATAC-seq experiments (Validation Experiment A) and primary T cell flow cytometry experiments (Validation Experiment B). Two gRNAs per RE were used. For primary CAR T cell CRISPRi screens, the same gRNA pool was used as for the IL2 validation screen, which included eight gRNAs per enhancer.

### Stable cell line generation

Stable cell lines were generated by electroporation via the Neon Transfection System (Thermo Fisher Scientific). Cells were electroporated using recommended parameters, recovered in fresh media for 3 d, selected with zeocin (Thermo Fisher Scientific) for 10 d and then analyzed by flow cytometry for BFP to confirm dCas9 cassette expression near 100% of cells.

### qPCR

Brilliant II SYBR Green qPCR Master Mix (Agilent Technologies) was used. Primers (Elim Biopharmaceuticals) were validated before use by examining the melt curve. Analysis was performed using the ΔΔCt method, relative to the housekeeping gene *ACTB* and NTC gRNA controls. For ATAC–qPCR, Jurkat ATAC-seq libraries were used as input to qPCR, and optimal primers were designed in RE peaks using the ATAC Primer Tool^83^; one biological replicate of ATAC-seq was used as input to ATAC–qPCR due to sample volume constraints.

### Lentivirus production

For cell line experiments, Lenti-X HEK293T cells were seeded on plates overnight to achieve 95% confluency at time of transfection and transfected with packaging plasmids psPAX2 (1.5 µg; Addgene, 12260) and pMD2.G (4.5 µg; Addgene, 12259) and viral expression vector (6 µg) per 10-cm plate using Opti-MEM (Gibco) and Lipofectamine 3000 transfection reagents (Thermo Fisher Scientific). Viral supernatant was collected at 48 h and concentrated using Lenti-X Concentrator (Clontech) following the manufacturer’s instructions, resuspended in cell culture media at 10× the original culture volume and stored at −80 °C.

For primary T cell experiments, similar steps were followed with the following modifications. Cells were seeded in Opti-MEM I Reduced Serum Medium with L-glutamine (Gibco) supplemented with 5% FBS, 1 mM sodium pyruvate (Gibco) and 1× non-essential amino acids (Gibco) (cOpti-MEM) in T25 flasks in 5 ml. Cells were transfected with psPAX2 (3.1 µg; Addgene, 12260), pMD2.G (1.5 µg; Addgene, 12259), expression vector (4.2 µg), Lipofectamine 3000 (20.1 µl) and P3000 (18.5 µl; Thermo Fisher Scientific) in 3.7 ml. At 6 h, media were replaced with cOpti-MEM supplemented with ViralBoost at 1:500 dilution (ALSTEM). Lentiviral supernatant was harvested 24 h and 48 h after transfection, centrifuged at 500*g* for 5 min at 4 °C to remove debris, concentrated with Lenti-X Concentrator and resuspended in Opti-MEM at 100× the original culture volume.

### Flow cytometry and fluorescence-activated cell sorting

All antibodies were used at 1:20–1:200 dilutions. All cells were stained in flow cytometry staining buffer (eBioscience). FlowJo (version 10.6.1) software was used for all analysis. Cells were analyzed by flow cytometry (Attune NxT, Thermo Fisher Scientific, or LSR II, BD Biosciences) or sorted based on stained markers and gRNA expression (GFP or mScarlet) (FACSAria II, BD Biosciences). Fluorescence-activated cell sorting (FACS) was performed at the Stanford Shared FACS Facility.

For Jurkat intracellular cytokine staining, cells were stained with Zombie NIR viability dye at 1:1,000 dilution in PBS at 10 million cells per 100 µl for 15 min at 4 °C, washed, fixed using Cyto-Fast Fix/Perm Buffer Set (BioLegend) for 25 min at 22 °C, washed and stored in Cyto-Last Buffer (BioLegend) at 4 °C in the dark for 1–3 d. Before sorting, fixed cells were permeabilized and stained with IL2-BV711 (BioLegend, clone MQ1-17H12, cat. no. 500346, lot no. B354636) and IFNG-APC (BioLegend, clone B27, cat. no. 506510, lot no. B329616) antibodies for 45 min at 22 °C, washed with fix/perm buffer and resuspended in staining buffer. For Perturb-seq, cells were similarly stained with Zombie NIR fixable viability dye. For Jurkat validation CRISPRi experiments, cells were stained with CD3E-BV785 (BioLegend, clone OKT3, cat. no. 317329, lot no. B311209) or CD47-BV605 (BioLegend, clone CC2C6, cat. no. 323119, lot no. B300088) antibodies.

For primary T cell flow cytometry experiments, cells were stained with Ghost Dye Red 780 (Tonbo Biosciences), CD4-BV510 (BioLegend, clone OKT4, cat. no. 317444) and CD8-PerCP/Cyanine5.5 (BioLegend, clone SK1, cat. no. 344710), fixed and permeabilized with BD Cytofix/Cytoperm (BD Biosciences), stained for intracellular IL2 with IL2-APC (BioLegend, clone MQ1-17H12, cat. no. 500310) as described for Jurkat T cells and analyzed by flow cytometry (Attune NxT, Thermo Fisher Scientific). Plots shown are for live gated cells from a culture of CD3^+^ T cells (from which CD4^+^ and CD8^+^ are gated) or pre-isolated memory CD4^+^ cells. For CD4^+^ and CD8^+^ cell analysis, data were normalized to NTC cells on a per-donor basis. For memory CD4^+^ cell analysis, data were normalized to NTC and unstimulated cells on a per-donor basis. Perturbation strength was calculated by additionally normalizing by normalized TSS percent IL2^+^ values on a per-donor basis. Jurkat validation flow cytometry data were analyzed similarly.

For primary T cell pooled gRNA screens, cells were stained with Ghost Dye Red 780 (Thermo Fisher Scientific), fixed and permeabilized with Cyto-Fast Fix/Perm Buffer Set (BioLegend) and stained for intracellular IL2 (BioLegend, clone MQ1-17H12, cat. no. 500346, lot no. B354636). CD4^+^ memory primary T cell phenotype was verified using the following cell surface markers: CD3-PE (BioLegend, clone UCHT1, cat. no. 300441); CD4-BV511 (BioLegend, clone OKT4, cat. no. 317444); CD8-PerCP/Cyanine5.5 (BioLegend, clone SK1, cat. no. 344710); CD45RA-BV711 (BioLegend, clone HI100, cat. no. 304138); CD45RO-FITC (BioLegend, clone UCHL1, cat. no. 304204); CD62L-PE/Cy7 (BioLegend, clone DREG-56, cat. no. 304822); and CCR7-BV421 (BioLegend, clone G043H7, cat. no. 353208).

### Pooled K562 and Jurkat screening

Cells were infected with lentivirus gRNA pools in polybrene (8 µg ml^−1^) at a multiplcity of infection (MOI) of 0.1 (K562) or 0.2 (Jurkat), as confirmed by flow cytometry for GFP or mScarlet expression on days 2 and 3 after infection. Dox (1 µg ml^−1^) was added at the time of infection or 6 d before the screen endpoint and refreshed every 24 h. For the K562 screen, cells were expanded for 6 d after infection and frozen in aliquots on day 6 in CryotStor CS10 (STEMCELL Technologies). Before sorting, cells were thawed and allowed to recover in culture in dox^+^ media for 18 h and then sorted for live, gRNA^+^ cells.

For Jurkat screens, 0.5 µg ml^−1^ puromycin (Thermo Fisher Scientific) was added on day 3 after infection, puromycin selected for 4 d and confirmed by flow cytometry to have near 100% gRNA expression. On day 7, dox induction was started and continued for 6 d. On day 13, cells were activated at approximately 2–4 million cells per millilter for 8 h using CD3 antibody (BioLegend, clone OKT3, cat. no. 317347, lot no. B338622) coated tissue culture plates and media containing dox (1 µg ml^−1^), CD28 antibody (3 µg ml^−1^; BioLegend, clone CD28.2, cat. no. 302943, lot no. B335272), PMA (1×), ionomycin (1×) and Brefeldin A (1×) (PMA/iono/BrefA were used from Cell Activation Cocktail, BioLegend). gRNA^+^ cell number (accounting for MOI) was maintained at 1,000× the number of gRNAs included in the gRNA pool throughout the screen. For the initial screen, the above steps were modified to begin dox induction at the time infection; puromycin selection was performed from day 3 to day 7; and the screen was stopped on day 7.

### Single-cell library preparation

Single and double perturbations were performed in separate single-cell captures. Sorted cells were prepared using the Chromium Next GEM Single Cell 5′ Kit v2, Chromium Next GEM Chip K Single Cell Kit and Library Construction Kit (10x Genomics), following the Chromium Next GEM Single Cell 5′ Reagent Kits v2 (Dual Index) with Feature Barcoding user guide (CG000330 Rev A).

GEX libraries were constructed as recommended. For gRNA detection, oligos complementary to each of the gRNA scaffolds (Sa and Sp) were spiked into the RT reaction at 11.43 pmol each.

Sa: AAGCAGTGGTATCAACGCAGAGTACacaagttgacgagataaacacgg

Sp: AAGCAGTGGTATCAACGCAGAGTACcgactcggtgccactttttc

For step 2.2, cDNA primers were used (green; 10x Genomics, PN 2000089) instead of feature cDNA primers (purple; 10x Genomics, PN 2000277). For step 2.3, GEX is in the pellet (2.3 A), and gRNAs are in the supernatant (2.3B); both portions were retained; and library construction was performed separately. gRNA library construction was performed using a custom PCR protocol, and Sa and Sp gRNA libraries were constructed separately. PCR1: outer nested PCR, F CTACACGACGCTCTTCCGATCT, R_sa acaagttgacgagataaacacgg, R_sp CGACTCGGTGCCACTTTTTC (98 °C for 3 min; 20 cycles at 98 °C for 20 s, 66 °C (Sa)/68 °C (Sp) for 30 s and 72 °C for 20 s; and 72 °C for 5 min). PCR2: inner nested PCR and adapter common region addition, F same primer as PCR1,

R_sa GTGACTGGAGTTCAGACGTGTGCTCTTCCGATCTgataaacacggcattttgccttg,

R_sp GTGACTGGAGTTCAGACGTGTGCTCTTCCGATCTcaagttgataacggactagcctt

(same cycling conditions as PCR1, with annealing temperatures 66 °C (Sa)/65 °C (Sp)). PCR3: sample index PCR, P5 and P7 Dual Index TT Set A (98 °C for 3 min; 15 cycles at 98 °C for 20 s, 54 °C for 30 s and 72 °C for 1 min; and 72 °C for 5 min). After each PCR, products were run on E-Gel EX 2% agarose and size selected.

### Design parameters for single-cell screens

CRISPRai was specifically designed to be highly scalable, and there is no inherent limitation on the number of perturbations CRISPRai can perform. Similar to direct capture Perturb-seq, CRISPRai screens have a tradeoff between the number of targets in the pool and the number of single cells that the user wants to assay at once. CRISPRai is highly scalable because we leverage the simultaneous direct capture of two gRNAs, which enables pooled cloning and virus production. Using current commercially available technologies, CRISPRai can be scaled to thousands of perturbations, as recently demonstrated by Replogle et al.^91^ for genome-scale direct capture Perturb-seq, which can be further expanded using emerging technologies. To maximize CRISPRai Perturb-seq data quality, we suggest the following: (1) analyze at least 40–50 cells per gRNA genotype; (2) anticipate approximately 50% efficacy of dual gRNA detection (for example, plan for sequencing 80–100 cells per gRNA to yield 40–50 cells with high-confidence gRNA detection) to accommodate the lower gRNA detection rate of two compared to one gRNA per cell; (3) include two or more gRNAs per gene to enable gRNA correlation analysis; and (4) incorporate single perturbation (CRISPRi and CRISPRa) controls to enable genetic interaction analysis and to identify which gene pairs are amenable to bidirectional control.

### CRISPR gRNA enrichment library preparation

Genomic DNA (gDNA) was extracted from sorted cells for different cytokine populations. Initial screen: IL2^+^IFNG^−^ (IL2), IFNG^+^IL2^−^ (IFNG), IL2^+^IFNG^+^(DP), IL2^−^IFNG^−^ (NEG) and unsorted (UN) cells. Validation screen: IL2^+^ (IL2), IL2^−^ (NEG) and UN cells. Cells were washed with PBS and resuspended in 1× lysis buffer (10 mM Tris pH 8, 5 mM EDTA, 0.5% SDS, 1× (0.4 mg ml^−1^) Proteinase K) (Thermo Fisher Scientific) in water at 10 million cells per 800 µl, incubated at 55 °C for 2 h and then 65 °C for 16–20 h overnight. Samples were then cooled to room temperature for 10 min, and Triton X-100 (Sigma-Aldrich) was added to a final concentration of 0.5%. The number of cells per population used for gDNA extraction was 0.2–15 million and 10–20 million for the initial and validation screens, respectively. For samples with more than 2 million sorted cells, gDNA was then purified using the Quick-DNA Miniprep Kit (Zymo Research), following the ‘Cell Suspensions and Proteinase K Digested Samples’ recommended protocol. For samples with fewer than 2 million cells, a precipitate-based method was used for gDNA extraction. After addition of Triton X-100 and sodium acetate to 10%, 2.5× volumes of 100% EtOH was added; samples were placed at −20 °C for 1 h followed by centrifugation at 20,000*g* for 15 min at 4 °C; the supernatant was removed; 75% EtOH was added; centrifugation was performed again; and pellets were dried overnight at room temperature and resuspended in elution buffer.

Library preparation from gDNA was performed by three PCR steps. PCR1: multiple reactions per sample were set up with 2 µg or less of gDNA with outer nested primers complementary to the gRNA cassette (98 °C for 3 min; 14 cycles of 98 °C for 20 s, 58 °C for 20 s and 72 °C for 40 s; and 72 °C for 2 min) and concentrated with DNA Clean & Concentrator (Zymo Research). PCR2: inner nested primers (98 °C for 30 s; six cycles of 98 °C for 15 s, 60 °C for 15 s and 72 °C for 45 s; and 72 °C for 2 min) and size selected using SPRI beads 0.75× cleanup. PCR3: Tru-seq-based indexing primers (98 °C for 30 s; six cycles of 98 °C for 15 s, 63 °C for 15 s and 72 °C for 45 s; and 72 °C for 2 min) and size selected using SPRI beads 0.75× cleanup. After each PCR, products were checked on E-Gel EX 2% agarose.

Primer sequences:

PCR1

mU6_outer_fw: cagcacaaaaggaaactcaccctaactgtaaag

sasgRNA_PCR_3Rev: tctcgccaacaagttgacgagataaaca

PCR2

p7_saRNA_stagger2_rev: GTGACTGGAGTTCAGACGTGTGCTCTTCCGATCTccttgttatagtagattctgtttccagagtactaTAAC

p7_saRNA_stagger1_rev: GTGACTGGAGTTCAGACGTGTGCTCTTCCGATCTcttgttatagtagattctgtttccagagtactaTAAC

p7_saRNA_stagger0_rev: GTGACTGGAGTTCAGACGTGTGCTCTTCCGATCTtgttatagtagattctgtttccagagtactaTAAC

p5_mU6_0nt_stagger: ACACTCTTTCCCTACACGACGCTCTTCCGATCTtcccttggagaaccaccttgt

p5_mU6_1nt_stagger: ACACTCTTTCCCTACACGACGCTCTTCCGATCTCtcccttggagaaccaccttgt

p5_mU6_2nt_stagger: ACACTCTTTCCCTACACGACGCTCTTCCGATCTGCtcccttggagaaccaccttgt

### ATAC-seq in Jurkat T cells

Jurkat CRISPRai T cells were transduced with individually cloned gRNAs (two gRNAs per RE) and processed under the same conditions as the Jurkat enhancer pooled screens. On the day of collection, cells were harvested for bulk ATAC-seq library preparation according to published protocols^99^. ATAC-seq reads were aligned to reference genome hg19 with Bowtie 2 (ref. ^100^) (version 2.3.4.1) using the parameter –very-sensitive. Data were filtered to remove mitochondrial reads, retain proper pairs (-f 0×2) and remove ambiguously mapped reads (MAPQ > 10, -q 10). BAM files were sorted and indexed with SAMtools (version 1.8). BedGraph coverage files were generated using bamCoverage from deepTools (version 3.3.1_py36)^101^ with parameters –numberOfProcessors 10–binSize 50–normalizeUsing CPM–region chr4. For quantification, data were further normalized by the total signal for chr4 per sample using a pseudocount of 1 × 10^−4^ and scaled to 1 × 10^6^.

### Primary T cell CRISPRi experiments and pooled screen

The CRISPRi plasmids used for primary T cell experiments were SFFV-ZIM3KRAB-dCas9-2A-mCherry or SFFV-ZIM3KRAB-dCas9-BlastR. To generate these plasmids, we replaced dCas9-VP64 on Lenti-SFFV-dCas9-VP64-2A-mCherry (Addgene, 180263) with ZIM3KRAB-dCas9 from Addgene, 154472, using Gibson assembly. The *ZIM3* KRAB domain was used. Next, mCherry was replaced with BlastR (Addgene, 52962) using Gibson assembly. The Lenti 1928z CAR construct was a gift from Dan Goodman. The high-affinity HA-GD2-28z CAR sequence was a gift from the Crystal Mackall laboratory^82^ and was cloned into the Lenti-1928z plasmid, replacing the 1928z CAR with the HA-GD2-28z CAR using Gibson assembly.

For all primary T cell experiments, cells were activated on day 0 using anti-human CD3/CD28 CTS Dynabeads (Thermo Fisher Scientific) at a 1:1 cell:bead ratio at 1 million cells per milliliter. Cells were transduced with each lentivirus sequentially after Dynabead activation: dCas9-KRAB at 18 h, CAR constructs at 26 h (when added) and gRNAs at 40 h. On day 9, cells were reactivated with ImmunoCult Human CD3/CD28/CD2 T Cell Activator (STEMCELL Technologies) with 6.25 μl ml^−1^ culture medium at 2 million cells per milliliter. One hour after reactivation, GolgiPlug Protein Transport Inhibitor (BD Biosciences) was added at a 1:1,000 dilution, and, after 7 h, cells were stained for cell surface proteins, fixed and permeabilized and stained for intracellular cytokines.

For arrayed primary T cell flow cytometry experiments, the above steps were followed with the following modifications. Fresh Leukopak cells were pre-enriched for CD3^+^ T cells using an EasySep Human T Cell Isolation Kit (STEMCELL Technologies) before experiments.

For pooled primary T cell screens, the above steps were followed with the following modifications. Fresh Leukopak cells were pre-enriched for CD4^+^ memory T cells using an EasySep Human Memory CD4^+^ T Cell Enrichment Kit (STEMCELL Technologies) before experiments. CD4^+^ memory T cell phenotype was verified by flow cytometry immediately after isolation using cell surface markers CD3, CD4, CD8, CD45RA, CD45RO, CD62L and CCR7. Cells were treated with 10 µg ml^−1^ blasticidin for 6 d starting on day 3 after activation. Cells were collected on day 9 and stained for live/dead and intracellular IL2. IL2^−^ and IL2^+^ populations were sorted by FACS; gDNA was isolated; and gRNA enrichment libraries were constructed as described for Jurkat T cell screens.

### Sequencing

Library quality was checked by Bioanalyzer (Agilent Technologies) and quantified by KAPA Library Quantification Kit (Roche). Sequencing was performed on a NovaSeq 6000 (Illumina, Novogene) or a NextSeq 550 (Illumina). For single-cell Perturb-seq libraries, libraries were sequenced at approximately 6,000 reads per cell for gRNA and approximately 30,000–50,000 reads per cell for GEX. For the Jurkat enhancer screens, gRNA enrichment libraries were sequenced at approximately 1.5 million reads per sample for the initial screen and approximately 7.5 million reads per sample for the validation screen (~1,200 reads per gRNA after filtering), and gRNA1 and gRNA2 in the double gRNA cassette were sequenced in R1 and R2 paired reads, respectively, and paired in silico. For primary T cell enhancer screens, gRNA enrichment libraries were sequenced at 2.5–3 million reads per sample (on average ~50,000 reads per gRNA and minimum ~2,400 reads per gRNA). For bulk Jurkat ATAC-seq, libraries were sequenced at more than 29 million reads per sample.

### Single-cell gRNA and transcriptome analysis

scRNA-seq reads were aligned to the GRCh38 reference genome and quantified using cellranger count (10x Genomics, version 5.0.0). CRISPR gRNA expression was quantified using cellranger count (10x Genomics, version 5.0.0) by specifying gRNA sequences and corresponding genes in features.csv. Downstream data analysis was done in R (version 3.6.1) using Seurat (version 3.2.3).

Data from five total captures were combined to one Seurat object. Cells were filtered: number of genes > 200, number of genes < 5,500–8,300, transcriptome unique molecular identifiers (UMIs) < 27,000–75,000, percent mitochondrial reads < 10%, detected gRNAs > 20 (background signal distribution) and gRNA UMIs > 50, with exact parameters differing for each capture for ranges listed above. gRNA labels for each cell were assigned based on cellranger feature calls. Only cells with one or two cellranger-detected gRNAs were retained for single gRNA and double gRNA captures, respectively. gRNA groups with fewer than 250 cells with target gene detected or with low cell numbers (*n* < 20) were removed. gRNA pools contained two gRNAs per gene for single perturbations and one gRNA per gene for double perturbations. For each gene, the gRNA with higher magnitude log_2_FC in the single perturbations was used for the double perturbation gRNA. If the two gRNAs for a given gene were not concordant in target gene log_2_FC expression for single perturbations, only the gRNA with greater magnitude change was retained for analysis. For these reasons, the following gRNAs were removed from the dataset: CEBPA.a1, CEBPA.a2, MAP2K3.a1, MYC.a1, MYC.a2, MYC.e1.a1, MYC.e1.a2, SPI1.a2, RIPK2.a2, ATF5.i1, CEBPB.i1 and FOSL1.i2. Gene expression was log normalized with a scaling factor of 1 × 10^4^. gRNA expression was normalized using relative counts with a scaling factor of 100. To quantify the number of gRNAs expressed per cell above a certain expression level threshold, we applied a threshold of 20% and 10% of total gRNA expression reads for cells expected to have single and double perturbations, respectively, and applied these thresholds to cells after filtering out cells without any gRNA expression and after filtering for quality control metrics described previously. We estimated gRNA detection false-negative rate (FNR, defined as true double but detected to be single) and false-positive rate (FPR, defined as true single but detected to be double) to be 48% and 29%, respectively, using non-filtered data. It should be noted that high FNRs are expected for single-cell data due to dropout. FPRs and FNRs can be corrected for by grouping single and double perturbation cells via cell hashing antibodies^88^ or separate captures that impart separate sample barcodes.

For Fig. 1, only gRNA groups with more than 40 cells were included. Differential expression for CRISPR target genes was performed FindMarkers() using normalized counts and a logistic regression model with batch as a latent variable. Batch was defined as the day on which 10x captures were performed, either day 1 or day 2. For Fig. 2, the top 2,000 most variable genes were found using variance stabilization transformation (vst). All genes were centered and scaled, and batch and percent mitochondrial reads were regressed out using ScaleData(). Principal component analysis (PCA) was performed on the top 2,000 most variable genes, followed by nearest neighbor graph construction, cluster determination using the original Louvain algorithm and UMAP dimensionality reduction using the top principal components (PCs). All further analyses were performed with regression on batch as the only latent variable except for UMAP reduction of 24,661 cells, which was regressed on batch and percent mitochondrial reads.

Next, the subset of cells with SPI1 and GATA1 gRNAs was retained, and variable gene selection was repeated. Perturbation-driven cells were identified as clusters that were not composed of equal representations from all gRNA groups. Non-perturbation-driven cells were removed, and variable feature selection, PCA, neighbor graph construction, clustering and UMAP reduction were performed again. All DE testing was performed on either all genes or genes in the indicated TF target gene sets using normalized counts and logistic regression with batch as a latent variable. For module score analysis, ENCODE TF target gene sets for SPI1 and GATA1 were downloaded from Harmonizome^70–72^, and genes were identified as being unique to either set or shared. Erythroid (*n* = 419, human bone marrow CD34 negative-selected and GYPA positive-selected erythroblasts, single-cell RNA-seq^68^) and myeloid (*n* = 394, human peripheral blood LIN(CD3, CD19, CD56)^−^CD14^+/lo^ monocytes, single-cell RNA-seq^69^) gene sets were obtained from the literature. Module scores were calculated using AddModuleScore() using normalized, scaled and batch-regressed counts. GO term enrichment was performed with clusterProfiler (version 3.14.0) enrichGO().

For all DE gene analysis, statistical significance was determined by genes passing *P*_adj < 0.05 and abs(log_2_FC) > 0.5. For analysis of regulatory modes for downstream target genes of SPI1 and GATA1, DE genes were filtered for statistical significance. Regulatory modes for downstream target genes were defined using the following thresholds (difference = log_2_FC observed − log_2_FC expected from additive model for double perturbation): synergy difference > 0.1 (greater magnitude than expected or opposite sign than expected) and buffer difference > 0.7 (lower magnitude than expected), and the remaining genes were classified as additive. For TF ChIP-seq analysis, the top 50 genes with the most additive phenotype were selected. The random gene subset was generated by randomly selecting 300 genes detected in the Perturb-seq experiment that were not contained in the SPI1 and GATA1 DE gene sets. For SPI1 and GATA1 TF ChIP-seq analysis, bigWig files containing ‘fold change over control’ were downloaded from ENCODE^70,71^: ENCFF080RWW, ENCFF838RXA and ENCFF334KVR (GATA1) and ENCFF172UZW, ENCFF454PTX and ENCFF216QNX (SPI1). log_2_FC was calculated with a pseudocount of 0.01. log_2_FC ChIP-seq signal of GATA1 or SPI1 within 1 kb of the promoter (2-kb window) or within ABC model enhancers for a given gene was calculated by taking the average signal across the RE. A gene was classified as being bound by a TF if one or more REs (including promoter or enhancers) had log_2_FC ChIP-seq signal > 5 (normalized to input).

All functions referenced above are from Seurat unless noted otherwise. Statistical testing was performed using stat_compare_means() from ggpubr or FindMarkers() and FindAllMarkers() from Seurat. All plots were generated in R using Seurat, ggplot2 (version 3.3.2), ggpubr (version 0.2.4) and pheatmap (version 1.0.12).

### CRISPR gRNA enrichment analysis

For Jurkat gRNA enrichment analysis, CRISPR gRNA enrichment reads were counted; dual gRNAs were paired in silico from paired-end reads; and a raw read counts per gRNA matrix was created using Python 3 (version 3.7.4). Downstream data analysis was done in R (version 3.6.1). gRNA pairs were filtered for pairs with the sum of raw read counts across all sorted populations > 300 reads. Reads were normalized per sample by dividing by the total reads per sample and scaling by 1 × 10^6^ and log2 transformed with a pseudocount of 1. Fold change was calculated between each population versus the cytokine-negative population. z-scores were computed by centering and scaling relative to the mean and standard deviation of all NTC gRNAs. z-scores were used for the majority of further analyses. z-scores were calculated independently for the initial and validation *IL2* locus screens. To ensure that the initial CRISPRai screens were benchmarked to positive control gRNAs with strong effects, the TSS gRNAs with the strongest effects were retained, and the following gRNAs were removed from the analysis: TSS.a.2 and TSS.i.2 (IL2) and TSS.a.1 and TSS.i.2 (IFNG). For the IL2 validation screen, the following gRNAs were removed from the analysis because they exhibited strong outlier behavior: NTC.a.1.val, TSS.a.3.val, TSS.i.1.val, or because it was not detected: E6.a.8.val.

Expected double gRNA enrichment was calculated by summing the log_2_FC gRNA enrichment z-scores of the corresponding single perturbations: log_2_FC z-score(single1) + log_2_FC z-score(single2). Residuals were calculated from the line of best fit between expected and observed double log_2_FC z-score. Pearson correlations were calculated using cor(). Perturbation strength was calculated through a second normalization step relative to TSS log_2_FC (E log_2_FC − TSS log_2_FC). log_2_FC difference was calculated through an alternate second normalization step relative to the average NTC log_2_FC (E log_2_FC − average NTC log_2_FC).

The genome-wide off-target analysis in Supplementary Table 7 was performed using the publicly available web tool from IDT (https://www.idtdna.com/site/order/designtool/index/CRISPR_SEQUENCE). For each targeting gRNA, the 20-bp 5′ of the PAM was uploaded in FASTA format to the web tool, generating a list of potential off-target sites genome wide, with associated metadata such as number of mismatches, genomic location and whether the off-target location overlaps a gene. We then intersected these results with the CRISPRi/a screening data of Schmidt et al.^50^ to evaluate whether any off-target sites overlapped genes known to impact IL2 or IFNG expression. For each identified potential off-target gene, we queried the Schmidt et al. screen hits to see if targeting that gene impacted expression of the relevant cytokine when targeted with the relevant guide type (that is, CRISPRi or CRISPRa). If the gene was a hit in any of the relevant conditions in Schmidt et al., we included the condition with the most significance (lowest false discovery rate (FDR)) into Supplementary Table 7. This analysis revealed that 14 of the 19,999 (0.07%) potential off-target sites analyzed overlapped with a gene that was a hit in a relevant condition of Schmidt et al., and 13 of our 204 targeting gRNAs (6.3%) contained at least one off-target site overlapping one of these genes. Thus, off-target overlap with coding genes is unlikely to play a major role in the observed efficacy of our gRNAs.

For histone ChIP-seq analysis, bigWig files containing ‘fold change over control’ were downloaded from ENCODE^70,71^. File accessions used were as follows: for activated T cells, ENCFF233LPC, ENCFF370YXG, ENCFF356ZKI, ENCFF704NYS, ENCFF741XLV, ENCFF158HYB, ENCFF232FZK, ENCFF206YVE, ENCFF336KWY, ENCFF164WIU, ENCFF060VND, ENCFF398QTX, ENCFF940OQY, ENCFF903VVJ, ENCFF356TWG, ENCFF248VJB, ENCFF690AHR, ENCFF243FBP, ENCFF624BMC and ENCFF352EYP and, for resting T cells, ENCFF906URN, ENCFF787PDH, ENCFF787LLC, ENCFF820GJE, ENCFF984ZEE, ENCFF829WQD, ENCFF055UPO, ENCFF459VQV, ENCFF041OBG, ENCFF543OQM, ENCFF863YFO, ENCFF896VDJ, ENCFF560YNU, ENCFF309ISK, ENCFF953MIX and ENCFF478JER. Regions overlapping each enhancer were used to estimate enhancer-specific histone signatures using GRanges and IRanges. For TF motif enrichment analysis, position frequency matrices (PFMs) were downloaded from JASPAR^85^:

JASPAR2022_CORE_vertebrates_non-redundant_pfms_jaspar.txt. TF motif score calculation in each enhancer was performed using matchMotifs() from ChromVar^97^ and motifmatchr^102^ using parameters genome = hg38, out = scores, bg = subject and p.cutoff = 5 × 10^−5^ and filtered for the top-scoring motifs.

For genome tracks, the following datasets were used. ABC model predictions used for tracks and all other ABC model analyses: AllPredictions.AvgHiC.ABC0.015.minus150.ForABCPaperV3.txt.gz^45^. For Jurkat cell type predicted enhancers, the ABC model uses Jurkat ATAC-seq and Jurkat H3K27ac ChIP-seq and mixed cell type Hi-C^4^. The following file accessions were downloaded from ENCODE^70,71^: H3K27ac-activated T cell ChIP-seq ENCFF370YXG; H3K27ac resting T cell ChIP-seq ENCFF787LLC; H3K4me3-activated T cell ChIP-seq ENCFF940OQY; H3K4me3 resting T cell ChIP-seq ENCFF863YFO; H3K4me1-activated T cell ChIP-seq ENCFF755MCS; H3K4me1 resting T cell ChIP-seq ENCFF041OBG; activated T cells DNase-seq ENCFF997BFO; and CTCF-activated T cell ChIP-seq ENCFF523IEI. H3K27ac resting Jurkat ChIP-seq (Gene Expression Omnibus (GEO) GSM1697882)^41^; BRD4 activated T cell ChIP-seq GSM5573170_Stim_BRD4.bw (GEO GSM5573170)^103^; JUNB and cFOS activated CD4 T cell ChIP-seq (GEO GSE116695; Sequence Read Archive (SRA) SRR7475866 and SRR7475865)^86^; RUNX1 resting Jurkat ChIP-seq (GEO GSM1697879)^41^; and resting Jurkat ATAC-seq (GEO GSM4130892)^104^. FASTQ files downloaded from the SRA were converted to bigWig files using Galaxy tools (https://galaxyproject.org/, version 22.05) and recommended pipelines^105^. Activated Jurkat ATAC-seq shown in tracks was generated for this manuscript using cells receiving NTC gRNAs.

For SRE score analysis, enhancer coordinates and SRE scores were downloaded from the Multiplexed CRISPRi EnhancerNet website (http://enhancer.stanford.edu/, not versioned)^30^ for the *IL2* gene in Jurkat T cells. For the subset of enhancers shared between our screen and the SRE dataset, SRE score was plotted for all enhancer pairs. The following enhancers were shared between the CRISPRai screen and the SRE dataset: E4, E5, E7, E8 and E9.

For genetic interaction analysis, we took the following approach. (1) Calculate expected double perturbation log_2_FC z-scores by summing the values of the single perturbations. (2) Fit a linear model to the relationship between expected and observed log_2_FC z-scores for double perturbations. (3) Calculate the residual between the linear model and observed double perturbation log_2_FC z-score. (4) Determine significance by using two methods as described below. For method 1, we determined which RE pairs are outside 1 s.d. from the mean of residuals and required that this ‘hit’ be shared by all three replicates, which yielded three significant enhancer pairs. For method 2, we checked the normality of the residual z-scores using a Shapiro–Wilk normality test, which gave *P* = 0.17, *P* = 0.65 and *P* = 0.40 for Rep1, Rep2 and Rep3, respectively, indicating that these follow a normal distribution; assuming normality, we calculated *P* values for each residual z-score using pnorm() and took a cutoff of *P* < 0.05 as significant (without multiple hypothesis correction), which yielded six, five and three significant enhancer pairs for Rep1, Rep2 and Rep3, respectively. To take a stringent approach, we took only RE pairs that were called significant by both methods to be true significant hits, which yielded three RE pairs, as all pairs passing method 1 criteria also passed method 2 criteria.

Primary T cell gRNA enrichment data were analyzed as described above for Jurkat gRNA enrichment data. As sequencing depth was high for all gRNAs, no pseudocount was added.

All plots were generated in R using ggplot2 (version 3.3.2), ggpubr (version 0.2.4) and pheatmap (version 1.0.12). Genome tracks were generated using rtracklayer (version 1.46.0) and Gviz (version 1.30.0). In all box plots, statistical analysis was performed using stat_compare_means() from ggpubr. Statistical significance was performed using a two-sided Wilcoxon test using wilcox.test() unless otherwise noted. *P* values were corrected for multiple hypothesis testing using the Benjamini–Hochberg procedure where indicated.

### Reporting summary

Further information on research design is available in the Nature Portfolio Reporting Summary linked to this article.

## Online content

Any methods, additional references, Nature Portfolio reporting summaries, source data, extended data, supplementary information, acknowledgements, peer review information; details of author contributions and competing interests; and statements of data and code availability are available at 10.1038/s41587-024-02213-3.

## Supplementary information

Supplementary Information Supplementary Fig. 1. Note: Supplementary Tables are included as a separate Excel file, as noted in the section below.

Reporting Summary

Supplementary Table Excel file with Supplementary Tables 1–7, each as a separate tab.

## Data Availability

All single-cell RNA-seq, single-cell CRISPR gRNA, CRISPR gRNA enrichment and Jurkat ATAC-seq data have been deposited in the Gene Expression Omnibus (https://www.ncbi.nlm.nih.gov/geo/query/acc.cgi?acc=GSE220976) under accession code GSE220976. Gene sets are available through the Molecular Signatures Database (https://www.gsea-msigdb.org/gsea/msigdb) and Harmonizome (https://maayanlab.cloud/Harmonizome/).

## Code availability

For single-cell analysis, gRNA counts were quantified using cellranger count, available at https://www.10xgenomics.com/support/software/cell-ranger/latest, and downstream analysis was performed using Seurat, available at https://satijalab.org/seurat/. No custom pipelines were developed for analysis.
