# Supplementary material for: Bidirectional epigenetic editing reveals hierarchies in gene regulation
Source: Nat Biotechnol. 2024 May 17;43(3):355–68. doi: 10.1038/s41587-024-02213-3 (PMC11569274; doi:10.1038/s41587-024-02213-3)
Supplement: Supplementary file 1 — Supplementary Fig. 1. Note: Supplementary Tables are included as a separate Excel file, as noted in the section below. [file 41587_2024_2213_MOESM1_ESM.pdf]

nature biotechnology

Article https://doi.org/10.1038/s41587-024-02213-3

Bidirectional epigenetic editing reveals hierarchies in gene regulation

In the format provided by the authors and unedited

Pacalin et al.
Bidirectional epigenetic editing reveals hierarchies in gene regulation
Supplementary Information

Table of Contents

Supplementary Figure 1
Supplementary Table 1
Supplementary Table 2
Supplementary Table 3
Supplementary Table 4
Supplementary Table 5
Supplementary Table 6
Supplementary Table 7

Supplementary Fig. 1: Optimization of Jurkat T cell activation

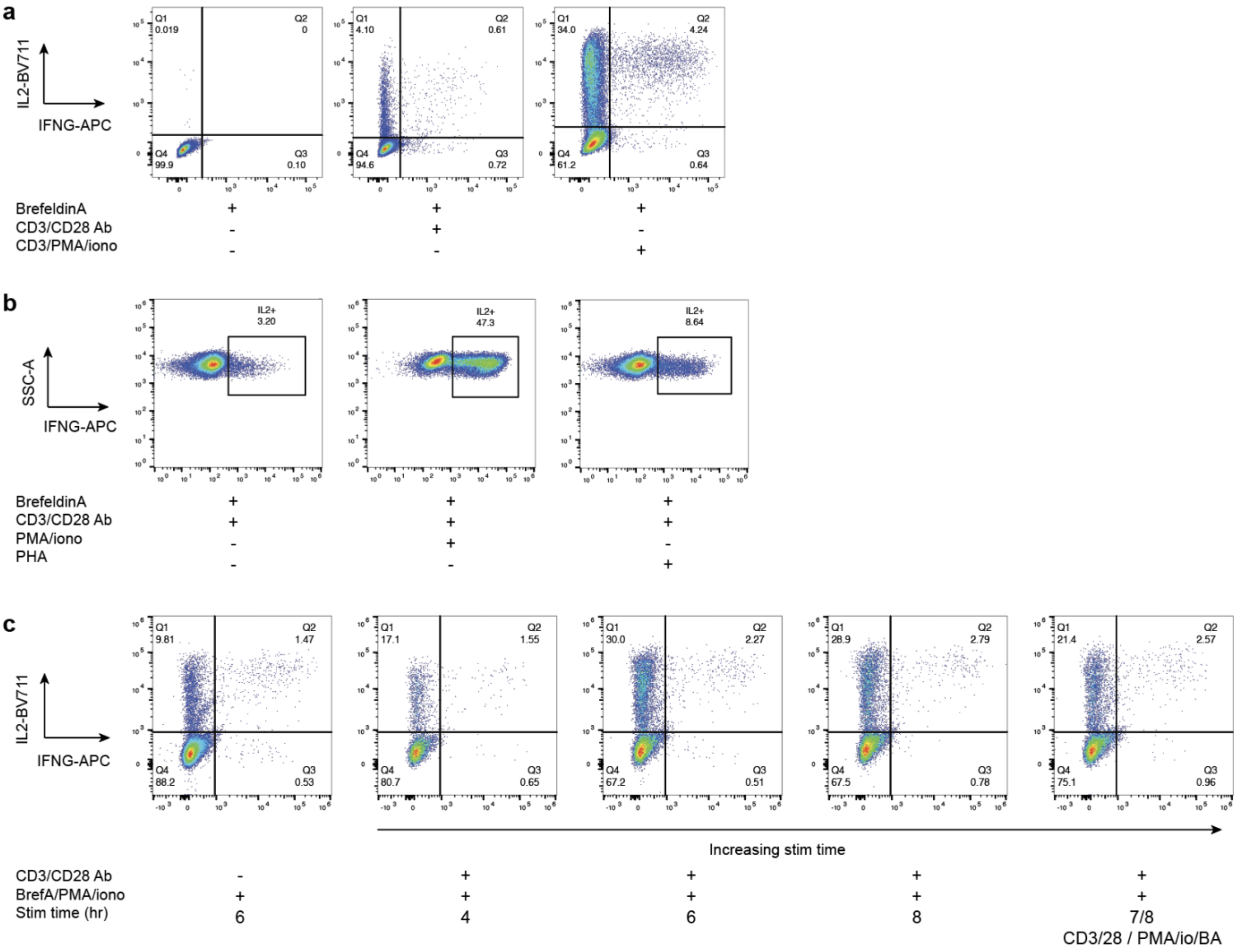

Supplementary Fig. 1 (related to Fig. 3-6). Optimization of Jurkat T cell activation. Intracellular cytokine staining by flow cytometry for IL2 and IFNG in Jurkat T cells in various conditions. **(a)** Comparison of CD3/CD28 and PMA/ionomycin for 10 hr activation. **(b)** Comparison of combinations of CD3/CD28, PMA/ionomycin, and PHA for 10 hr activation. **(c)** Time course of CD3/CD28 and PMA/ionomycin activation. **(a-c)** n=1 biological replicate.
