# Supplementary material for: Bidirectional epigenetic editing reveals hierarchies in gene regulation
Source: Nat Biotechnol. 2024 May 17;43(3):355–68. doi: 10.1038/s41587-024-02213-3 (PMC11569274; doi:10.1038/s41587-024-02213-3)
Supplement: Supplementary file 2 — Reporting Summary [file 41587_2024_2213_MOESM2_ESM.pdf]

nature portfolio

Corresponding author(s): Howard Y Chang

Last updated by author(s): Mar 14, 2024

Reporting Summary

Nature Portfolio wishes to improve the reproducibility of the work that we publish. This form provides structure for consistency and transparency in reporting. For further information on Nature Portfolio policies, see our Editorial Policies and the Editorial Policy Checklist.

Statistics

For all statistical analyses, confirm that the following items are present in the figure legend, table legend, main text, or Methods section.

n/a	Confirmed	
☐	☒	The exact sample size (*n*) for each experimental group/condition, given as a discrete number and unit of measurement
☐	☒	A statement on whether measurements were taken from distinct samples or whether the same sample was measured repeatedly
☐	☒	The statistical test(s) used AND whether they are one- or two-sided<br>*Only common tests should be described solely by name; describe more complex techniques in the Methods section.*
☐	☒	A description of all covariates tested
☐	☒	A description of any assumptions or corrections, such as tests of normality and adjustment for multiple comparisons
☐	☒	A full description of the statistical parameters including central tendency (e.g. means) or other basic estimates (e.g. regression coefficient) AND variation (e.g. standard deviation) or associated estimates of uncertainty (e.g. confidence intervals)
☐	☒	For null hypothesis testing, the test statistic (e.g. *F*, *t*, *r*) with confidence intervals, effect sizes, degrees of freedom and *P* value noted<br>*Give P values as exact values whenever suitable.*
☒	☐	For Bayesian analysis, information on the choice of priors and Markov chain Monte Carlo settings
☒	☐	For hierarchical and complex designs, identification of the appropriate level for tests and full reporting of outcomes
☐	☒	Estimates of effect sizes (e.g. Cohen's *d*, Pearson's *r*), indicating how they were calculated

Our web collection on statistics for biologists contains articles on many of the points above.

Software and code

Policy information about availability of computer code

Data collection	Single cell RNA-seq were aligned to the GRCh38 reference genome and quantified using cellranger count (10x Genomics, version 5.0.0). CRISPR gRNA expression was quantified using cellranger count (10x Genomics, version 5.0.0) by specifying gRNA sequences and corresponding genes in features.csv. CRISPR gRNA enrichment reads were counted, dual gRNAs were paired in silico from paired end reads, and a raw read counts per gRNA matrix was created using python3 (version 3.7.4). ATAC-seq reads were aligned to the hg19 reference genome using Bowtie2 (version 2.3.4.1), filtered to remove mitochondrial reads, retain proper pairs, and remove ambiguously mapped reads. Bam files were sorted and indexed with Samtools (version 1.8). Bedgraph coverage files were generated using bamCoverage from deepTools (version 3.3.1_py36).
Data analysis	Downstream data analysis and plotting was done in R (version 3.6.1), using ggplot2 (version 3.3.2), ggpubr (version 0.2.4), pheatmap (version 1.0.12), rtracklayer (version 1.46.0), Gviz (version 1.30.0). Single cell RNA-seq and CRISPR gRNA data was analyzed using Seurat (version 2.3.4). Gene Ontology (GO) term enrichment analysis was performed using clusterProfiler (version 3.14.0). ChIP-seq fastq files were used to generate BigWig files using Galaxy tools (galaxyproject.org, version 22.05). ChIP-seq bigWig files were downloaded from ENCODE. gRNA off-target analysis was performed using the web tool from IDT (https://www.idtdna.com/site/order/designtool/index/CRISPR_SEQUENCE, not versioned). FlowJo (version 10.6.1) was used for flow cytometry analysis.

For manuscripts utilizing custom algorithms or software that are central to the research but not yet described in published literature, software must be made available to editors and reviewers. We strongly encourage code deposition in a community repository (e.g. GitHub). See the Nature Portfolio guidelines for submitting code & software for further information.

Data

Policy information about availability of data

All manuscripts must include a data availability statement. This statement should provide the following information, where applicable:

- Accession codes, unique identifiers, or web links for publicly available datasets
- A description of any restrictions on data availability
- For clinical datasets or third party data, please ensure that the statement adheres to our policy

All single-cell RNA-seq, single-cell CRISPR gRNA, CRISPR gRNA enrichment, and ATAC sequencing data have been deposited in the Gene Expression Omnibus (GEO) under accession GSE220976. All other relevant data are available from the corresponding authors upon reasonable request.

Activity-by-Contact Model data was obtained from AllPredictions.AvgHiC.ABC0.015.minus150.ForABCPaperV3.txt.gz. JUNB and cFOS activated CD4 T cell ChIP-seq (GEO GSE116695, SRA SRR7475866 and SRR7475865). For SRE score analysis, enhancer coordinates and SRE scores were downloaded from the Multiplexed CRISPRi EnhancerNet website (enhancer.stanford.edu). ChIP-seq bigWig files were downloaded from ENCODE (accession numbers listed below and in Methods). gRNA off-target analysis was performed using the web tool from IDT (https://www.idtdna.com/site/order/designtool/index/CRISPR_SEQUENCE, not versioned).

ENCODE data for histone ChIP-seq data: ENCFF233LPC ENCFF370YXG ENCFF356ZKI ENCFF704NYS ENCFF741XLV ENCFF158HYB ENCFF232FZK ENCFF206YVE ENCFF336KWY ENCFF164WIU ENCFF060VND ENCFF398QTX ENCFF940OQY ENCFF903VVJ ENCFF356TWG ENCFF248VJB ENCFF690AHR ENCFF243FBP ENCFF624BMC ENCFF352EYP and for resting T cells ENCFF906URN ENCFF787PDH ENCFF787LLC ENCFF820GJE ENCFF984ZEE ENCFF829WQD ENCFF055UPO ENCFF459VQV ENCFF041OBG ENCFF543OQM ENCFF863YFO ENCFF896VDJ ENCFF560YNU ENCFF309ISK ENCFF953MIX ENCFF478JER. For GATA1: ENCFF080RWW, ENCFF838RXA, ENCFF334KVR. For SPI1: ENCFF172UZW, ENCFF454PTX, ENCFF216QNX.

Data used for genome tracks: H3K27ac activated T cell ChIP-seq ENCFF370YXG, H3K27ac resting T cell ChIP-seq ENCFF787LLC, H3K4me3 activated T cell ChIP-seq ENCFF940OQY, H3K4me3 resting T cell ChIP-seq ENCFF863YFO, H3K4me1 activated T cell ChIP-seq ENCFF755MCS, H3K4me1 resting T cell ChIP-seq ENCFF041OBG, activated T cells DNase-seq ENCFF997BFO, CTCF activated T cell ChIP-seq ENCFF523IEI. H3K27ac resting Jurkat ChIP-seq (GEO GSM1697882); BRD4 activated T cell ChIP-seq GSM5573170_Stim_BRD4.bw (GEO GSM5573170); JUNB and cFOS activated CD4 T cell ChIP-seq (GEO GSE116695, SRA SRR7475866 and SRR7475865), RUNX1 resting Jurkat ChIP-seq (GEO GSM1697879); resting Jurkat ATAC-seq (GEO GSM4130892).

Gene sets are available through MSigDb (gsea-msigdb.org/gsea/msigdb) and Harmonizome (maayanlab.cloud/Harmonizome/).

Human research participants

Policy information about studies involving human research participants and Sex and Gender in Research.

Reporting on sex and gender	Not applicable.
Population characteristics	Not applicable.
Recruitment	Not applicable.
Ethics oversight	Not applicable.

Note that full information on the approval of the study protocol must also be provided in the manuscript.

Field-specific reporting

Please select the one below that is the best fit for your research. If you are not sure, read the appropriate sections before making your selection.

☒ Life sciences ☐ Behavioural & social sciences ☐ Ecological, evolutionary & environmental sciences

For a reference copy of the document with all sections, see nature.com/documents/nr-reporting-summary-flat.pdf

Life sciences study design

All studies must disclose on these points even when the disclosure is negative.

Sample size	No statistical method was used to determine the sample size. For single-cell RNA and CRISPR gRNA experiments, the cell number per gRNA group are consistent with prior single-cell studies. We focused our single-cell analysis on cell groups with greater than 50 cells. This benchmark is based on the practice of published studies that use single-cell RNA-seq (Gasperini et al. Cell 2019, Xie et al. Molecular Cell 2017). For CRISPR gRNA enrichment experiments, we used 2 gRNAs for primary screens and 8 gRNAs for the validation screen per enhancer, which is comparable to published CRISPR screens (Horlbeck et al. Cell 2018, Gasperini et al. Cell 2019, Xie et al. Molecular Cell 2017, Bodapati et al. Genome Biology 2020).
Data exclusions	gRNAs that were not detected, did not have sufficient cell numbers or whose target gene was expressed below detection limits in single-cell experiments, or were not strongly not concordant with other gRNAs for the same target were examined and ultimately excluded from the analysis. These criteria were predetermined and are listed in Methods.

Replication	Single-cell sequencing performed across multiple 10x Genomics captures. CRISPR gRNA enrichment experiments were replicated in 2-3 biological replicates, which were concordant. ATAC-seq was performed in 2 biological replicates. qPCR data was replicated at least twice. Other experiments were replicated 2-3 times. All attempted replicates of ATAC-seq, qPCR, and gRNA enrichment screens were successful.
Randomization	Covariates, specifically batch of sample processing (day 1 or day 2) and percent mitochondrial reads per cell were controlled for during statistical testing of differential gene expression for single-cell RNA-seq analysis. This practice is standard for published work with this data type.
Blinding	Investigators were not blinded to group allocations due to personnel constraints.

Reporting for specific materials, systems and methods

We require information from authors about some types of materials, experimental systems and methods used in many studies. Here, indicate whether each material, system or method listed is relevant to your study. If you are not sure if a list item applies to your research, read the appropriate section before selecting a response.

Materials & experimental systems

n/a	Involved in the study
☐	☒ Antibodies
☐	☒ Eukaryotic cell lines
☒	☐ Palaeontology and archaeology
☒	☐ Animals and other organisms
☒	☐ Clinical data
☒	☐ Dual use research of concern

Methods

n/a	Involved in the study
☒	☐ ChIP-seq
☐	☒ Flow cytometry
☒	☐ MRI-based neuroimaging

Antibodies

Antibodies used	For validation CRISPRi experiments, cells were stained with CD3E-BV785 (Biolegend, clone OKT3, cat. no. 317329, lot no. B311209) or CD47-BV605 antibodies (Biolegend, clone CC2C6, cat. no. 323119, lot no. B300088). For gRNA enrichment screens, intracellular cytokines were stained with IL2-BV711 (Biolegend clone MQ1-17H12, cat. no. 500346, lot no. B354636) and IFNG-APC (Biolegend, clone B27, cat. no. 506510, lot no. B329616). Jurkat T cells were activated using CD3 antibody (Biolegend, clone OKT3, cat. no. 317347, lot no. B338622) and CD28 antibody (3ug/ml, Biolegend, clone CD28.2, cat. no. 302943, lot no. B335272). For primary T cell flow cytometry experiments, cells were stained with CD4-BV510 (Biolegend clone OKT4, cat. no. 317444), and CD8-PerCP/Cyanine5.5 (Biolegend clone SK1, cat. no. 344710), IL2-APC (Biolegend clone MQ1-17H12, cat. no. 500310). Memory CD4+ primary T cell phenotype was verified using CD3-PE (Biolegend clone UCHT1, cat. no. 300441), CD4-BV511 (Biolegend clone OKT4, cat. no. 317444), CD8-PerCP/Cyanine5.5 (Biolegend clone SK1, cat. no. 344710), CD45RA-BV711 (Biolegend clone HI100, cat. no. 304138), CD45RO-FITC (Biolegend clone UCHL1, cat. no. 304204), CD62L-PE/Cy7 (Biolegend clone DREG-56, cat. no. 304822), CCR7-BV421 (Biolegend clone G043H7, cat. no. 353208). All antibodies were used at 1:20 to 1:200 dilutions.
Validation	All antibodies were validated by the manufacturer directly in human peripheral blood mononuclear cells. Manufacturer validation includes specificity testing in 1-3 target cell types in single or multi color analysis, intensity testing by MFI, and QC testing with a series of titration dilutions. Antibodies were compared to no stain controls.

Eukaryotic cell lines

Policy information about cell lines and Sex and Gender in Research

Cell line source(s)	Lenti-X HEK293T were obtained from Clontech. K562 (CCL-238) and Jurkat (Clone E6-1, TIB-152) cell lines were obtained from ATCC. Primary bulk CD3+ T cells and isolated memory CD4+ T cells were were sourced from PBMC-enriched leukapheresis products (Leukopaks, STEMCELL Technologies) from healthy donors, after institutional review board–approved informed written consent.
Authentication	Cell lines were not authenticated, except for the isolated memory CD4+ T cells which were verified to express expected markers by flow cytometry.
Mycoplasma contamination	Cell lines were tested periodically for mycoplasma and found to be negative.
Commonly misidentified lines (See ICLAC register)	No commonly misidentified cell lines were used.

Flow Cytometry

Plots

Confirm that:

☒ The axis labels state the marker and fluorochrome used (e.g. CD4-FITC).

☒ The axis scales are clearly visible. Include numbers along axes only for bottom left plot of group (a 'group' is an analysis of identical markers).

☒ All plots are contour plots with outliers or pseudocolor plots.

☒ A numerical value for number of cells or percentage (with statistics) is provided.

Methodology

Sample preparation	For single-cell RNA-seq experiments, cryopreserved cells in Cryotstor CS10 (StemCell Technologies) were thawed for sorting. For CRISPR gRNA enrichment experiments, cells were used fresh from cell culture. All cells were washed with flow cytometry staining buffer (eBioscience) prior to staining. For validation CRISPRi experiments, cells were stained for 30min at 4C. For sorting, all cells for Perturb-seq and Jurkat gRNA enrichment screens were stained with Zombie NIR fixable viability dye at 1:1000 dilution in PBS (Biolegend). For primary cell flow cytometry experiments, cells from culture were stained with Ghost Dye Red 780 (Tonbo Biosciences), CD4 (Biolegend), and CD8 (Biolegend), fixed and permeabilized with BD Cytofix/Cytoperm (BD Biosciences), stained for intracellular IL2 (Biolegend). For primary T cell sorting, cells from culture were stained with Ghost Dye Red 780 (Thermo Fisher), fixed and permeabilized with Cyto-Fast™ Fix/Perm Buffer Set (Biolegend), stained for intracellular IL2 (Biolegend).
Instrument	All samples were sorted using a FACSAria II. All flow cytometry analysis that did not require sorting was performed using a BD LSRII or Attune Nxt.
Software	Flow cytometry data was analyzed using FlowJo (version 10.6.1).
Cell population abundance	Post-sort purities were confirmed to be > 95% and were validated by single cell RNA-sequencing data, CRISPR gRNA enrichment data, and live dead cell counting on Countess II.
Gating strategy	For single-cell experiments, cells were first gated on FSC/SSC, then gated to exclude doublets on FSC-A/FSC-W and SSC-A/SSC-W. Dead cells were excluded based on Zombie NIR viability staining. Cells were gated to include the top 70-75% of BFP expressing cells (high dCas9 expression), then gRNA+ cells (GFP+ or mSclarlet+) were sorted. For gRNA enrichment experiments, cells were first gated on FSC/SCC, then gated to exclude doublets on FSC-A/FSC-H and/or SSC-H/SSC-W. Dead cells were excluded based on Zombie NIR or Ghost Dye Red 780 viability staining. Cells containing gRNA were gated (mScarlet +), and cytokine populations were sorted. For primary Jurkat screens, sorted populations were NEG (IL2- IFNG-), IL2+ (IL2+ IFNG-), IFNG+ (IL2- ING+), and double positive (DP, IL2+ IFNG+). For validation screens, IFNG staining was not used, and sorted populations were NEG (IL2-) and IL2+. For primary T cell experiments, a similar gating strategy was used as described above on a population of CD3+ (from which CD4+ and CD8+ cells are gated) or isolated memory CD4+ T cells.

☒ Tick this box to confirm that a figure exemplifying the gating strategy is provided in the Supplementary Information.
